# Tumor-derived CXCL5 promotes human colorectal cancer metastasis through activation of the ERK/Elk-1/Snail and AKT/GSK3β/β-catenin pathways

**DOI:** 10.1186/s12943-017-0629-4

**Published:** 2017-03-29

**Authors:** Jingkun Zhao, Baochi Ou, Dingpei Han, Puxiongzhi Wang, Yaping Zong, Congcong Zhu, Di Liu, Minhua Zheng, Jing Sun, Hao Feng, Aiguo Lu

**Affiliations:** 1 Shanghai Minimally Invasive Surgery Center, Ruijin hospital, Shanghai Jiaotong University School of Medicine, Shanghai, People’s Republic of China; 2 Shanghai Institute of Digestive Surgery, Shanghai, People’s Republic of China; 3 Department of General, Visceral, Transplantation, and Vascular Thoracic Surgery, Hospital of University of LMU Munich, Munich, Germany; 4 Department of thoracic surgery, Ruijin hospital, Shanghai Jiaotong University School of Medicine, Shanghai, People’s Republic of China

**Keywords:** CXCL5, Colorectal cancer, EMT, Snail, β-catenin

## Abstract

**Background:**

Metastasis is a major cause of death in human colorectal cancer patients. However, the contribution of chemokines in the tumor microenvironment to tumor metastasis is not fully understood.

**Methods:**

Herein, we examinined several chemokines in colorectal cancer patients using chemokine ELISA array. Immunohistochemistry was used to detect expression of CXCL5 in colorectal cancer patients tissues. Human HCT116 and SW480 cell lines stably transfected with CXCL5, shCXCL5 and shCXCR2 lentivirus plasmids were used in our in vitro study. Immunoblot, immunofluorescence and transwell assay were used to examine the molecular biology and morphological changes in these cells. In addition, we used nude mice to detect the influence of CXCL5 on tumor metastasis in vivo.

**Results:**

We found that CXCL5 was overexpressed in tumor tissues and associated with advanced tumor stage as well as poor prognosis in colorectal cancer patients. We also demonstrated that CXCL5 was primarily expressed in the tumor cell cytoplasm and cell membranes, which may indicate that the CXCL5 was predominantly produced by cancer epithelial cells instead of fibroblasts in the tumor mesenchyme. Additionally, overexpression of CXCL5 enhanced the migration and invasion of colorectal cancer cells by inducing the epithelial-mesenchymal transition (EMT) through activation of the ERK/Elk-1/Snail pathway and the AKT/GSK3β/β-catenin pathway in a CXCR2-dependent manner. The silencing of Snail and β-catenin attenuated CXCL5/CXCR2-enhanced cell migration and invasion in vitro. The elevated expression of CXCL5 can also potentiate the metastasis of colorectal cancer cells to the liver in vivo in nude mice intrasplenic injection model.

**Conclusion:**

In conclusion, our findings support CXCL5 as a promoter of colorectal cancer metastasis and a predictor of poor clinical outcomes in colorectal cancer patients.

**Electronic supplementary material:**

The online version of this article (doi:10.1186/s12943-017-0629-4) contains supplementary material, which is available to authorized users.

## Background

Colorectal cancer (CRC) is one of the most common cancers worldwide [1]. Although many breakthroughs in the diagnosis and treatment of CRC have been made over the past few decades, CRC-related mortality remains high [2]. As the major cause of death for most cancer patients, tumor metastasis is an important adverse factor in the treatment and prognosis of CRC patients [3]. Tumor metastasis is a complicated and multistep process which requires the ability of the tumor to migrate and invade [4, 5]. Despite an increase in our understanding of cell biology and the identification of many metastasis-related molecules [6], the chemokine-related alterations in the tumor microenvironment that promote CRC metastasis remain largely unknown.

Chemokines are a multifunctional superfamily of small proteins that bind to G protein-coupled receptors on target cells. CXCL5 belongs to a subset of CXC chemokines and functions as the ligand for CXCR2 [7]. By binding to CXCR2, CXCL5 mediates various cellular behaviors including neutrophil trafficking, tumor cell migration and invasion [8]. Recent studies have reported that TNF-α-induced mesenchymal stromal cells (MSCs) can secrete CXCL5 to recruit CXCR2+ neutrophils that promote breast cancer metastasis [9]. These findings indicate that CXCL5 can act as a protumoral molecule in a paracrine way through recruiting immune cells. However, the mechanisms underlying the role of CXCL5 on CRC remain unknown.

In our study, we examined the expression of several chemokines in CRC tissues and found that CXCL5 was highly expressed in CRC tissues. In addition, we investigated the influence of CXCL5 on the survival of CRC patients and showed that CXCL5 levels were negatively correlated with prognosis. Furthermore, we also revealed that CXCL5 secreted by tumor cells was able to promote CRC migration through ERK/Elk-1/Snail-mediated EMT (Epithelial mesenchymal transition) and invasion via the AKT/GSK3β/β-catenin/MMP7 pathway in a CXCR2-dependent manner. Our study conclusively demonstrates that the overexpression of CXCL5 in CRC is able to promote CRC metastasis and predicts a poor outcome in patients with CRC, indicating that CXCL5 may serve as a potential therapeutic target.

## Results

### Chemokine expression profiles in CRC tissue

**Fig. 1:**
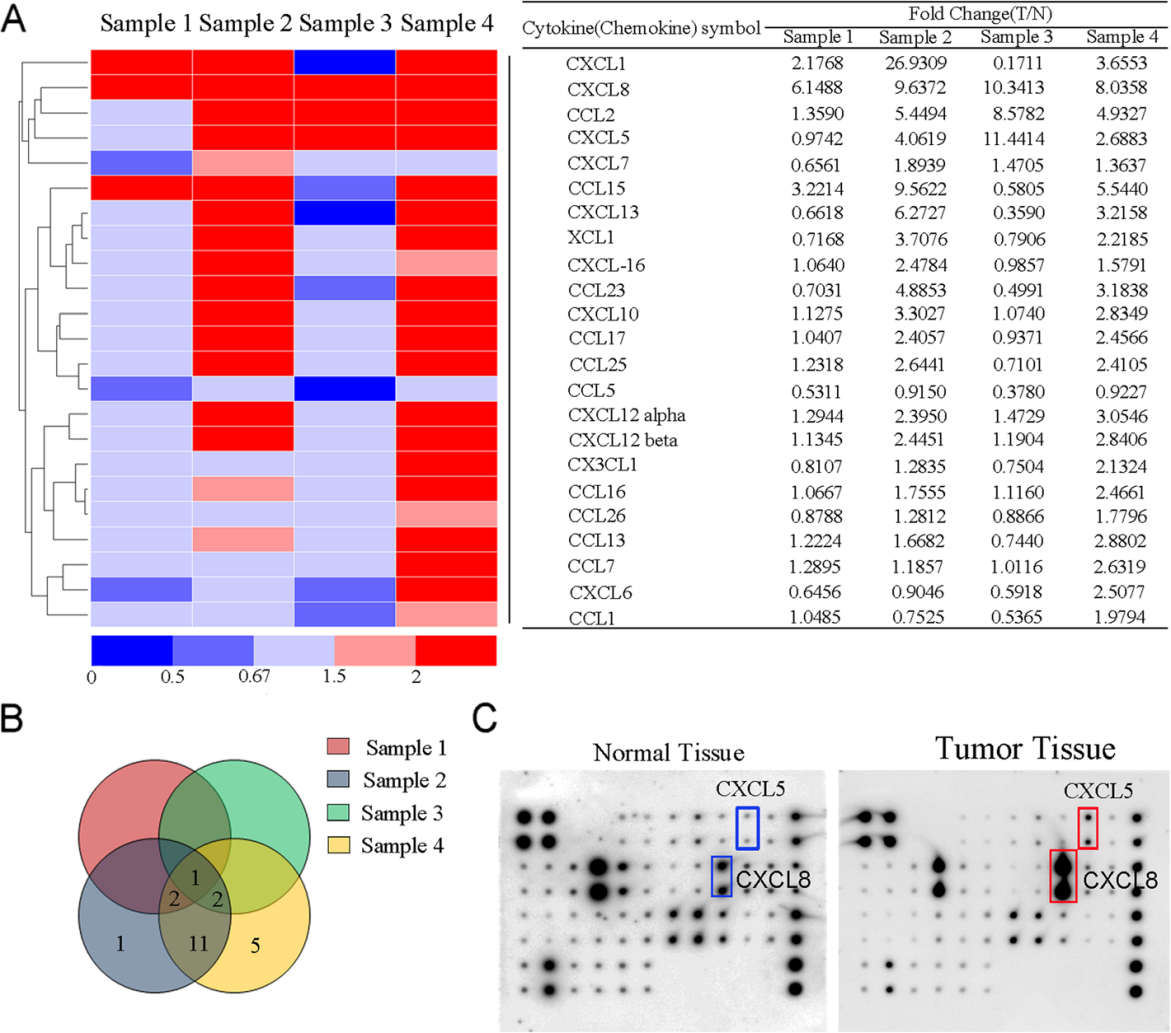
Expression profiles of chemokines in four CRC tissues. **a** A heat map displays the hierarchical clustering of the chemokine expression profiles. **b** An overlapping pie chart displays the distribution of chemokines in the four samples. **c** The protein microarray shows the expression of CXCL5, which was the primary focus of this study. The representative image is from sample 3. These four patient samples are selected according to their pathological stages which rages from Dukes I to Dukes IV. Pathological stages of the four tissues tested are, respectively: sample 1, Dukes I; sample 2, Dukes III; sample 3, Dukes II; sample 4, Dukes IV

To understand the expression profiles of the chemokines in CRC, we compared the distinct changes in several chemokines between the tumor and peritumoral normal tissues using a chemokine ELISA array. By setting a threshold of at least a 2-fold upregulation, the number of upregulated chemokines ranged from 3 (Sample 1) to 18 (Sample 4) in the various samples (Fig. 1a, b and c). This may be due to the different pathological stages of these samples (Sample 1:I vs Sample 4:IV). The number of upregulated chemokines increased with tumor progression. Five chemokines (CXCL8, CXCL1, CXCL5, CCL2, and CCL15) were upregulated in more than three samples (Fig. 1a). The association between CXCL8, CXCL1 and tumor metastasis has been thoroughly described in previous studies [10, 11]. Therefore, we primarily focused on the function of CXCL5.

### CXCL5 is upregulated in human CRC tissue

**Fig. 2:**
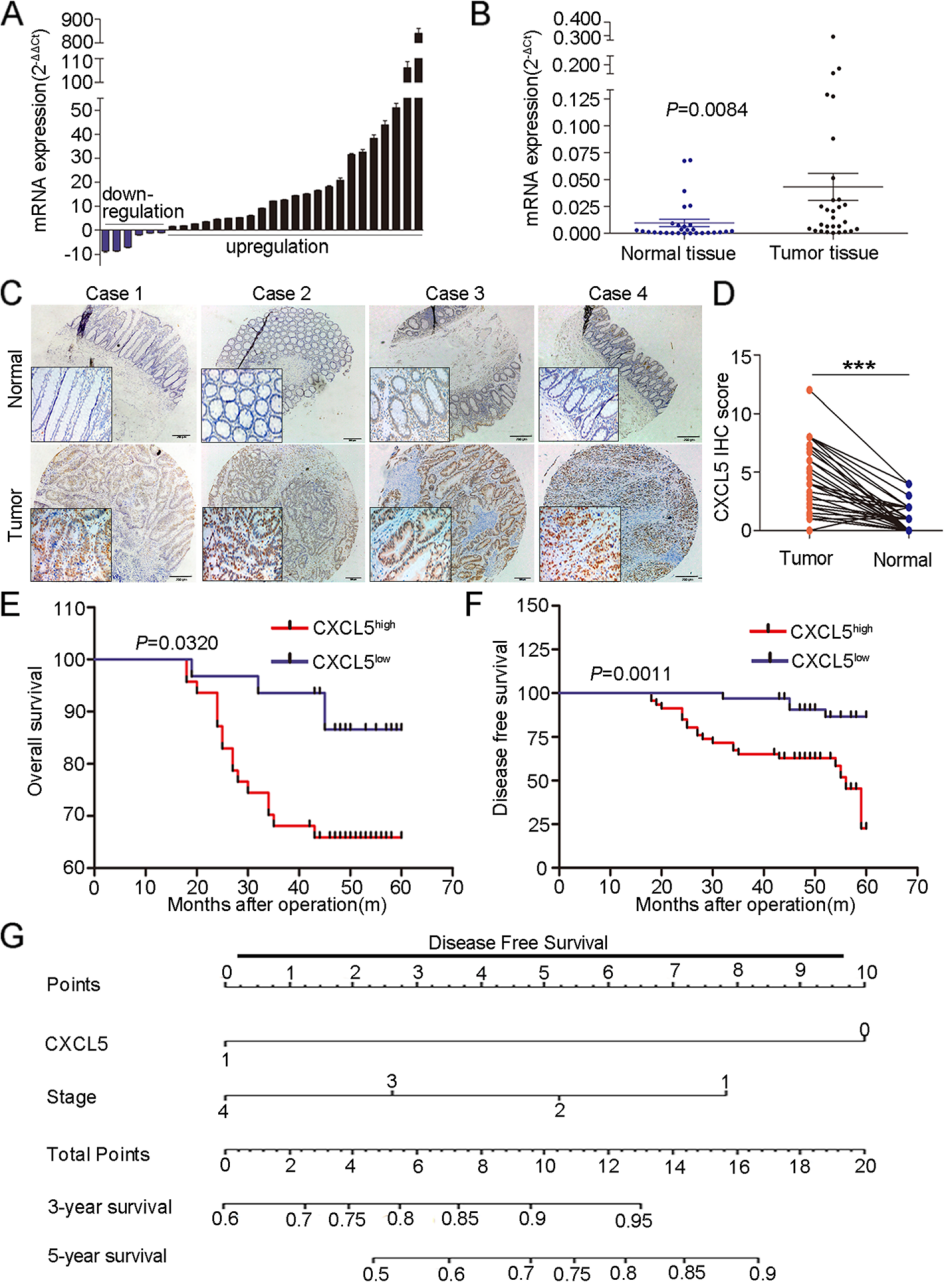
Increased expression of CXCL5 in CRC tissues, Kaplan-Meier analysis and nomogram plots. **a** & **b** RT-PCR results showing high expression of CXCL5 in CRC tissues. Data are showed by 2^-ΔΔCt^ (**a**) and 2^-ΔCt^ (**b**). **c** & **d** Representative immunohistochemistry images showing that CXCL5 is highly expressed in 61.5% of the cancer tissues in tissue microarray (tissues used are different from Figs. 1 and 2a & b). Scale: 200 μm. ***P<0.001. **e** & **f** Kaplan-Meier analysis of OS and DFS. The CXCL5^high^ group has a worse prognosis in OS and DFS compared with the CXCL5^low^ group. **g** Nomogram to predict DFS 3 and 5 years after surgery. CXCL5 line: 1 represents positive staining; 0 represents negative staining. Stage line: 1, 2, 3 and 4 represent Dukes stage I, II, III and IV. Calculation: find their points on the “point line” according to the CXCL5 staining and Dukes stage. Total points are equal to CXCL5 points plus Dukes stage points. The 3- or 5-year survival rates are indicated on the “3-year survival” line or “5-year survival” line according to their total points. For example, for a patient with CXCL5 positive staining and Dukes II. CXCL5 point is 0, Stage point is 5.2, total point is 5.2, 3-year survival prediction is 78%, 5-year survival prediction is 52%

The preliminary ELISA results verified that CXCL5 is upregulated in CRC tissues (Fig. 1). To confirm the increased expression of CXCL5 in human CRC tissues, we examined the mRNA and protein levels of CXCL5 in 29 paired CRC samples. In total, either 23 or 22 patients had high relative expression levels of CXCL5 in mRNA or protein level (Fig. 2a; Additional file 1: Figure S1A). The CXCL5 mRNA levels in the tumors were significantly higher than those in the peritumoral normal tissues (Fig. 2b). The expression levels of CXCL5 were also examined in tissue microarrays including 78 pairs of CRC samples. We selected a staining score of 4.5 as the cut-off value using X-tile software (Additional file 2: Figure S2). In this cohort, CXCL5 expression was upregulated in approximately 61.5% (48/78) of the paired tissues (Additional file 3: Table S4, Fig. 2c and d). Moreover, the expression of CXCL5 was primarily localized to the tumor cells rather than the mesenchymal portion of the cancerous tissue (Fig. 2c).

### High expression of CXCL5 is correlated with poor prognosis in CRC patients

We next analyzed the associations between CXCL5 expression and clinical features of CRC patients. The analysis revealed that the high expression of CXCL5 was significantly correlated with tumor size (*P* = 0.047), Dukes’ stage (*P* = 0.003), tumor invasion (*P* = 0.009), lymph node localization (*P* = 0.002), and liver metastasis (*P* = 0.022) (Additional file 3: Table S4). Kaplan-Meier survival analysis demonstrated that CXCL5^high^ patients had a poor overall survival (OS) compared to those with low CXCL5 expression (*P* = 0.032) (Fig. 2e). In addition, CRC patients with increased expression of CXCL5 were less likely to reach disease-free survival (DFS) status than CXCL5^low^ patients (*P* = 0.001) (Fig. 2f). The univariate COX regressive analysis indicated that CXCL5 was associated with OS (*P* = 0.019) and DFS (*P* = 0.004). However, the multivariate analysis showed that CXCL5 and Dukes’ Stage were independent prognostic factor for DFS but not for OS (Additional file 3: Table S5). Based on these results, we constructed a nomogram to predict DFS 3 and 5 years after surgery for CRC patients by integrating all of the significant independent factors for DFS (Fig. 2g). The calibration plot for the predicted probability of DFS 3 or 5 years after surgery revealed an optimal consistency between the prediction by nomogram and the actual survival rate (Additional file 1: Figure S1B and C).

Together, these results demonstrate that CXCL5 is associated with CRC progression and metastasis. Moreover, CXCL5^high^ patients have a poor prognosis, and CXCL5 is an independent prognostic factor of DFS.

### Tumor-derived CXCL5 promotes CRC cell migration via EMT in a CXCR2-dependent manner

**Fig. 3:**
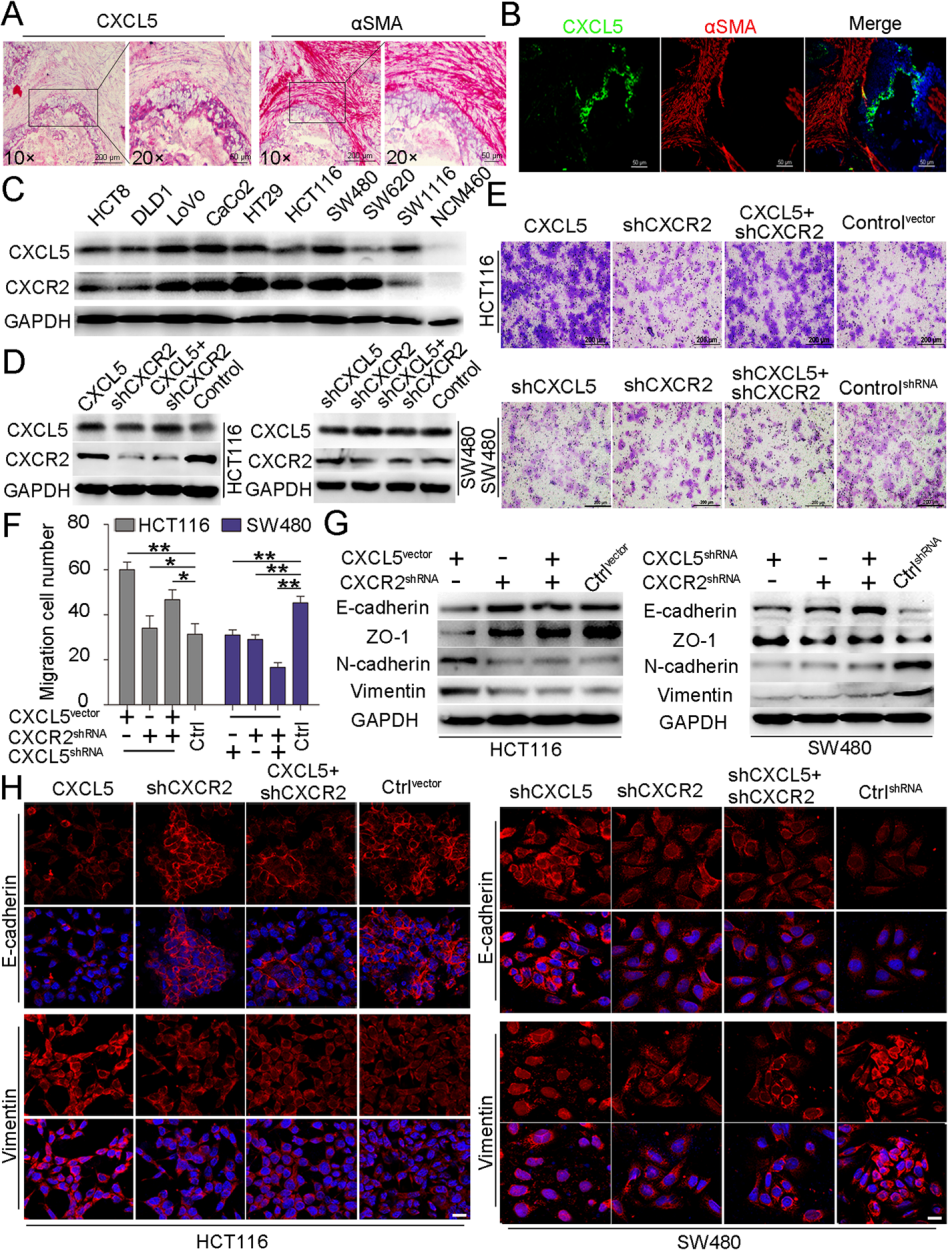
CXCL5 localization in CRC tissues, the expression of CXCL5 in CRC cell lines and CXCL5/CXCR2 promotes CRC cell migration through the induction of EMT. **a** & **b** Immunohistochemistry and immunofluorescence images showing the location of CXCL5 and αSMA (marker of fibroblasts) in CRC specimens. CXCL5 is mainly expressed in tumor tissue instead of mesenchymal tissue. **c** Immunoblots examining CXCL5 expression levels in CRC cell lines and normal colon epithelial cell. **d** Representative data from the immunoblot analysis verifying the changes of CXCL5 or CXCR2 using a CXCL5-vector or shRNA-inhibition of CXCL5 and CXCR2. **e** & **f** Representative images from the transwell migration assay in indicated CRC cells. Magnification, 200×. Scale, 200 μm. Data represent the mean ± SD. *P < 0.05, **P < 0.01, ***P < 0.001. **g** Immunoblot analysis of EMT marker expression in indicated cells. **h** Immunofluorescence images showing the changes in E-cadherin and Vimentin after different treatments with CXCL5-vector or shRNA-inhibition of CXCL5 and CXCR2. Scale, 50 μm

Fibroblasts are regarded as key determinants in the malignant progression of cancer through the secretion of various chemokines, including CXCL5 [12]. However, we found that CXCL5 was primarily secreted by cancer cells in CRC. In the IHC assay described above, we observed CXCL5 staining primarily in cancer cells. By counterstaining the fibroblasts, we further found that, although CXCL5 staining was observed in the tumor mesenchyme, CXCL5 was primarily expressed in the tumor lesions rather than the fibroblasts, which were visualized with the fibroblast marker α-smooth muscle actin (αSMA) (Fig. 3a and b). These results indicate that CXCL5 is primarily secreted by cancer cells. To further investigate this phenomenon, we examined the expression of CXCL5 and its receptor CXCR2 in various CRC cell lines. An elevated expression of CXCL5 was observed in the CRC cell lines compared with the normal colon epithelial NCM460 cell line. CXCL5 was highly expressed in SW480, as well as CaCo2 cells, and a low expression was observed in the SW620 and HCT116 cells (Fig. 3c). The levels of CXCL5 in the supernatants of the CRC cell lines as detected by ELISA were consistent with the results of the immunoblots (Additional file 4: Figure S3A). All four cell lines had high levels of CXCR2 (Fig. 3c), and we chose the HCT116 and SW480 cell lines for further investigation. Stable ectopic expression and down-regulation of CXCL5, as well as knock-outs of CXCR2, were induced in the HCT116 and SW480 cells, and expression was confirmed by immunoblotting (Fig. 3d) and immunofluorescence staining (Additional file 4: Figure S3B).

We next investigated the biological changes of cells with high and low expression of CXCL5 and found that the ectopic expression of CXCL5 in HCT116 cells (HCT116^CXCL5^), as well as the down-regulated expression of CXCL5 in SW480 cells (SW480^shCXCL5^), either promoted (in HCT116^CXCL5^) or inhibited (in SW480^shCXCL5^) the migration of these two cell lines compared with the control group. Additionally, the inhibition of CXCR2 in HCT116 cells (HCT116^shCXCR2^) suppressed the ability to migrate and also reversed migration-promoting behavior in HCT116^CXCL5^ cells (HCT116^CXCL5-shCXCR2^). Similarly, the inhibition of CXCR2 in SW480 cells (SW480^shCXCR2^) and SW480^shCXCL5^ cells (SW480^shCXCL5-shCXCR2^) suppressed their migration (Fig. 3e and f). These results indicate that the function of CXCL5 is dependent on CXCR2. Moreover, we also observed morphological changes in the CRC cells. As shown in Additional file 4: Figure S3C, HCT116^CXCL5^ acquired a mesenchymal, spindle-like morphology compared with the HCT116^shCXCR2^, HCT116^CXCL5-shCXCR2^ and control cells. These spindle-like characteristics were also observed in the SW480^shRNA^ cells, which had a high metastatic potential. However, the SW480^shCXCL5^, SW480^shCXCR2^ and SW480^shCXCL5-shCXCR2^ cells developed an epithelial cell-like morphology and tended to accumulate in clusters. Therefore, we hypothesized that CXCL5/CXCR2 promotes CRC cell migration by inducing EMT. To confirm our hypothesis, we examined changes in EMT markers in these cells. The results demonstrated that the upregulation of CXCL5 in HCT116^CXCL5^ cells led to lower expression of the epithelial markers E-cadherin and ZO-1 and higher expression of the mesenchymal markers N-cadherin and Vimentin (Fig. 3g and h). However, lower levels of E-cadherin and ZO-1, as well as an upregulation of N-cadherin and Vimentin, were observed in SW480^shRNA^ cells (Control^shRNA^), which is a highly metastatic CRC cell line. Elevated levels of E-cadherin and ZO-1, as well as reduced expression of N-cadherin and Vimentin, were also observed in the SW480^shCXCL5^, SW480^shCXCR2^ and SW480^shCXCL5-shCXCR2^ cells (Fig. 3g and h). These results indicate that CXCL5 is able to promote CRC cell migration by inducing EMT in a CXCR2-dependent manner.

### The CXCL5/CXCR2 axis induces EMT in CRC cells via the ERK/Elk-1 pathway

**Fig. 4:**
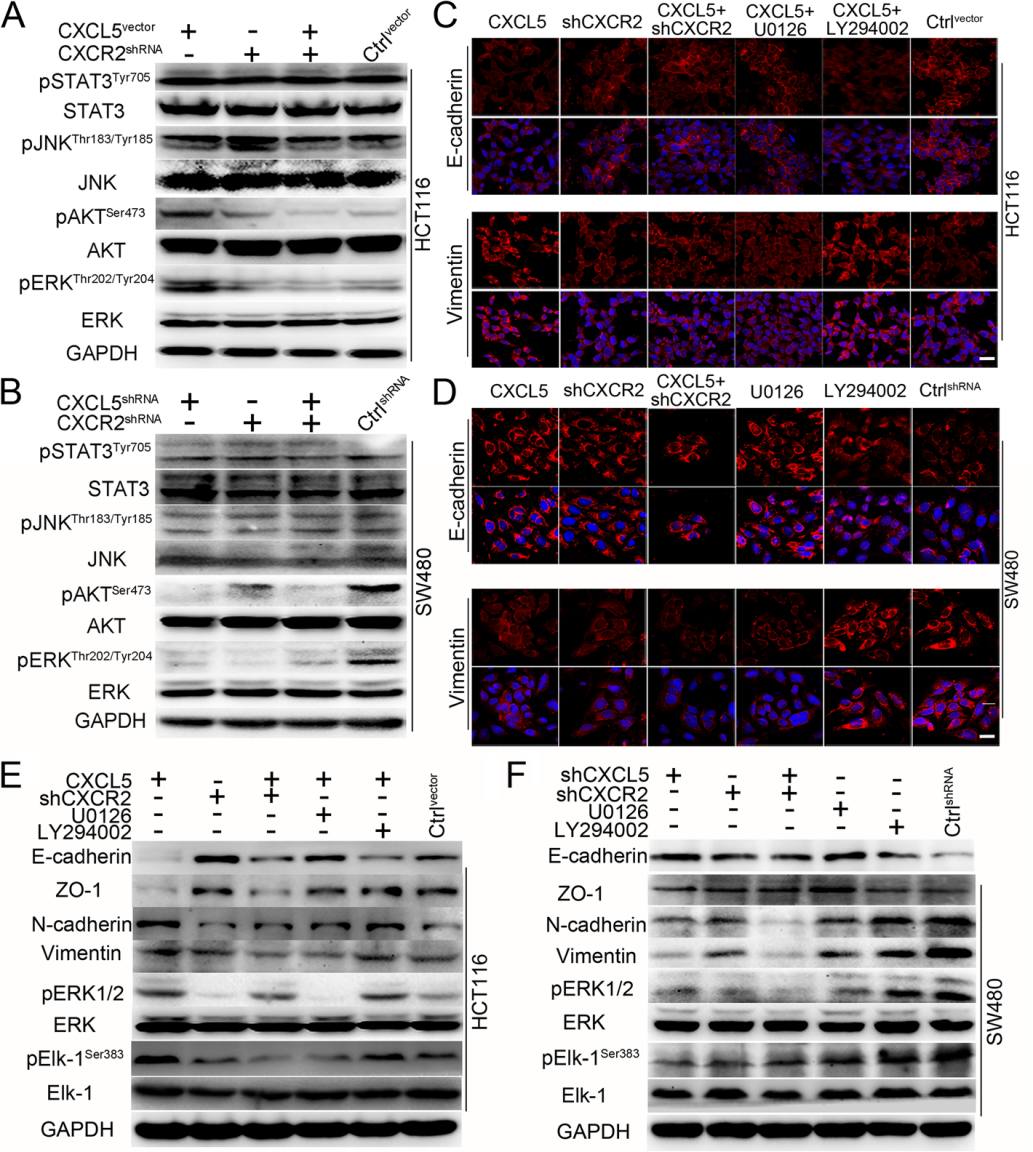
CXCL5/CXCR2 induces EMT in CRC cells via the ERK/Elk-1 pathway. **a** & **b** CXCL5 induces the phosphorylation of ERK and Akt in CRC cells in a CXCR2-dependent manner. No differences are observed in STAT3 and JNK pathway. **c** & **d** Representative immunofluorescence images showing that inhibition of the ERK pathway using U0126 upregulates E-cadherin expression and downregulates Vimentin expression in HCT116^CXCL5^ and SW480^shRNA^ cells. Inhibition of the AKT pathway using LY294002 is not able to change expression of E-cadherin or Vimentin in HCT116^CXCL5^ and SW480^shRNA^ cells. Scale, 50 μm. **e** & **f** Immunoblots showing that inhibition of the ERK pathway using U0126 upregulates E-cadherin expression and downregulates Vimentin expression in HCT116^CXCL5^ and SW480^shRNA^ cells. Inhibition of the AKT pathway using LY294002 is not able to change the expression of E-cadherin or Vimentin in HCT116^CXCL5^ and SW480^shRNA^ cells. Changes in Elk-1 are consistent with changes in ERK1/2

Previous studies have shown that CXCR2 can be activated by CXCL5 or other ligands to stimulate cancer progression through the activation of downstream pathways, such as JAK/STAT3, JNK, PI3K/AKT and ERK1/2 pathway [13, 14]. To analyze the mechanisms underlying the induction of EMT by CXCL5/CXCR2, we examined the activity of these pathways. As shown in Fig. 4a and b, ectopic CXCL5 expression in HCT116^CXCL5^ cells activated the ERK and AKT pathways, as indicated by the upregulation of phosphorylated ERK1/2 (pERK1/2) and AKT (pAKT^Ser473^). Similarly, pERK and pAKT were also highly expressed in SW480^shRNA^ cells. As expected, low levels of pERK and pAKT were observed in the SW480^shCXCL5^ cells. Additionally, the knock-down of CXCR2 inhibited the phosphorylation of ERK and AKT. Furthermore, the effects of CXCL5 were inhibited in the HCT116^CXCL5-shCXCR2^ cells. These results indicated that the phosphorylation of ERK and AKT was dependent on CXCR2. Moreover, CXCL5 had no effect on the activation of pSTAT3^Tyr705^ and pJNK^Thr183/Tyr185^.

Next, we examined which of these two CXCL5-activated pathways was able to induce EMT. We used inhibitors of the ERK pathway (U0126) and the AKT pathway (LY294002) to block the activity of these two pathways. Our results demonstrated that the inhibition of ERK pathway rather than AKT pathway changed the cellular morphology and reversed the effects of CXCL5 on migration in HCT116^CXCL5^ and SW480^shRNA^ cells (Additional file 5: Figure S4). Based on these findings, the effects of these two pathways on EMT markers were further evaluated using immunofluorescence and immunoblotting. The results revealed that suppression of the ERK pathway promoted the expression of E-cadherin and ZO-1, as well as downregulated the expression of N-cadherin and Vimentin. However, inhibition of the AKT pathway had little effect on these changes (Fig. 4e and f). This is consistent with the results of immunofluorescence (Fig. 4c and d). The downstream protein Elk-1 has been confirmed to be an effector of the ERK pathway [15], so we next investigated alterations of this effector. Our results showed that Elk-1 phosphorylation (pElk-1^Ser383^) either increased or decreased with the upregulation or downregulation of pERK in HCT116^CXCL5^ and SW480^shRNA^ cells, and the effect was blocked after the knock-down of CXCR2. Meanwhile, inhibition of the ERK pathway decreased the levels of pElk-1, which indicates that pElk-1 may act as downstream effector of the ERK pathway (Fig. 4e and f). Altogether, these results indicate that the CXCL5/CXCR2 axis is capable of promoting CRC cell migration by inducing EMT. The mechanism behind this primarily involves the ERK/Elk-1 pathway.

### Snail is involved in the CXCL5/CXCR2-mediated promotion of CRC cell migration and EMT and is regulated by the CXCL5/CXCR2 axis via the ERK/Elk-1 pathway

**Fig. 5:**
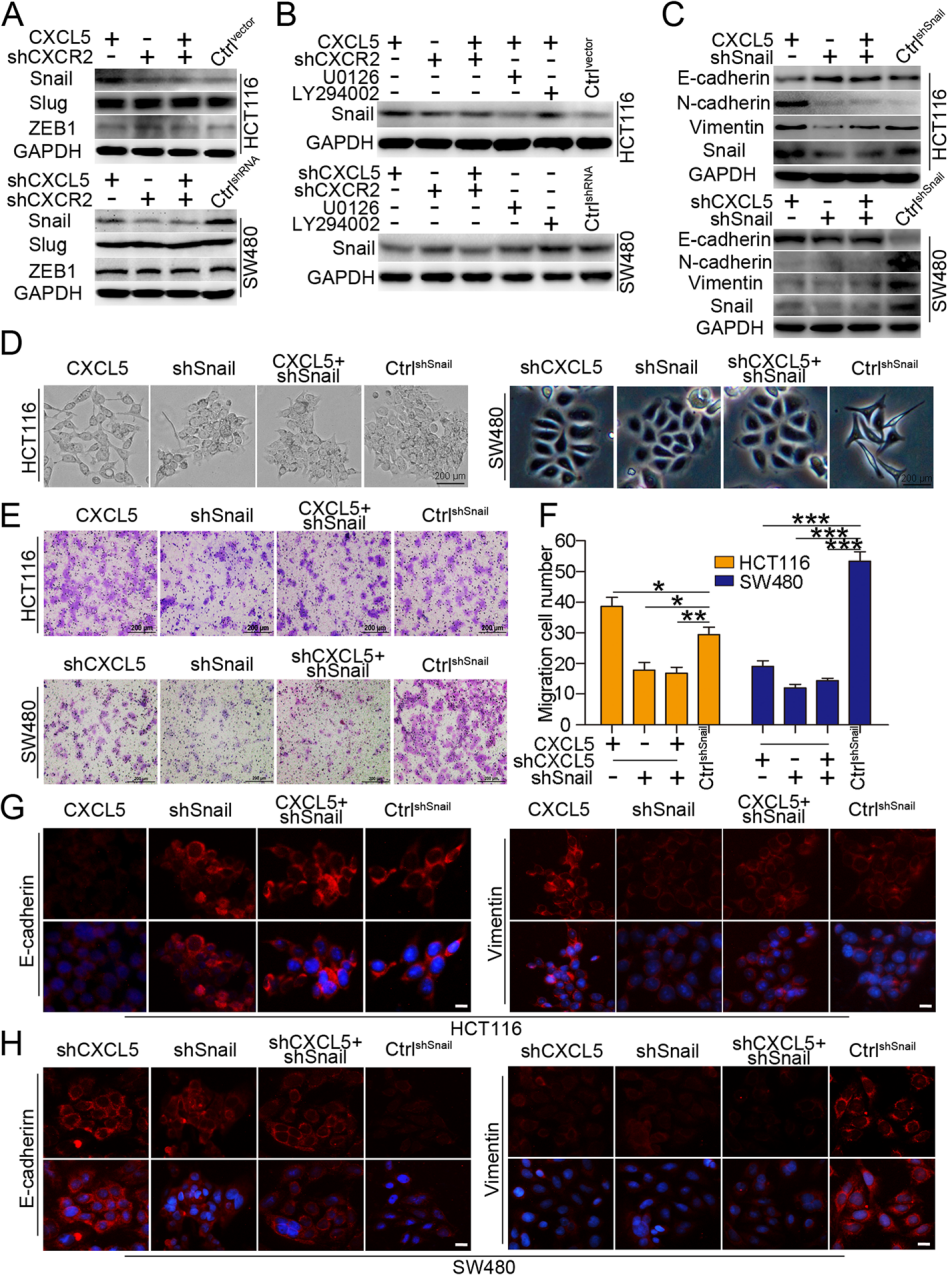
Snail is critical to CXCL5/CXCR2-mediated CRC migration. **a** Immunoblot analysis of Snail, Slug and ZEB1 levels in the indicated cells. CXCL5 is able to induce Snail expression. **b** Inhibition of the ERK pathway (U0126) instead of the AKT pathway (LY294002) downregulates Snail expression in the indicated cells. **c** Downregulation of Snail inhibits CXCL5-induced downregulation of E-cadherin and upregulation of Vimentin and N-cadherin in HCT116. Downregulation of Snail upregulates E-cadherin expression and downregulates Vimentin and N-cadherin expression in SW480. **d** Inhibition of Snail altered the morphology of cells in both the HCT116^CXCL5^ and SW480 groups. **e** & **f** Inhibition of Snail decreased the number of migrating cells in the indicated cell lines. Data are presented as the mean ± SD. *P < 0.05, **P < 0.01, ***P < 0.001. **g** & **h** Immunofluorescence images show that downregulation of Snail inhibits CXCL5-induced downregulation of E-cadherin and upregulation of Vimentin and N-cadherin in HCT116. Downregulation of Snail upregulates E-cadherin expression and downregulates Vimentin and N-cadherin expression in SW480. Scale, 50 μm

Previous studies have demonstrated that transcription factors, including Snail, Slug and ZEB1, which act as E-cadherin repressors, are involved in controlling the EMT transition [16, 17]. Consequently, to evaluate whether CXCL5/CXCR2-mediated EMT was regulated by these transcription factors, we assessed their expression profiles. Immunoblot data showed a significant increase in Snail levels in HCT116^CXCL5^ cells and a decrease in Snail levels in SW480^shCXCL5^, SW480^shCXCR2^ and SW480^shCXCL5-shCXCR2^ cells. However, there were no obvious alterations in Slug and ZEB1 expression (Fig. 5a). We next investigated the influence of Snail on EMT markers. The knock-down of Snail increased E-cadherin expression and downregulated N-cadherin and Vimentin levels in both HCT116 and SW480 cells. Snail shRNA also reversed the effect of CXCL5 in HCT116^CXCL5^ (Fig. 5c, g and h). Additionally, we observed a reversal of the epithelial cell-like morphological change and an increase of migrational cell number in the Snail-shRNA-transfected wild-type HCT116 and HCT116^CXCL5^ cells. An opposite effect was observed in SW480 groups (Fig. 5d, e and f). Altogether, the above results support a causal role for Snail in the CXCL5-induced and CXCR2-dependent EMT process. Additionally, we also found that the level of Snail was inversely downregulated in the U0126-HCT116^CXCL5^ group. Consistently, the use of U0126 in the SW480 group also decreased the expression of Snail. Furthermore, there were no obvious changes in Snail levels in the AKT pathway inhibitor groups of both the HCT116 and SW480 cell lines (Fig. 5b). Taken together, these results suggest that the CXCL5/CXCR2 axis induces EMT to promote CRC cell migration via the ERK/Elk-1/Snail pathway.

### The CXCL5/CXCR2 axis promotes CRC cell invasion through the AKT/GSK3β/β-catenin/MMP7 pathway

**Fig. 6:**
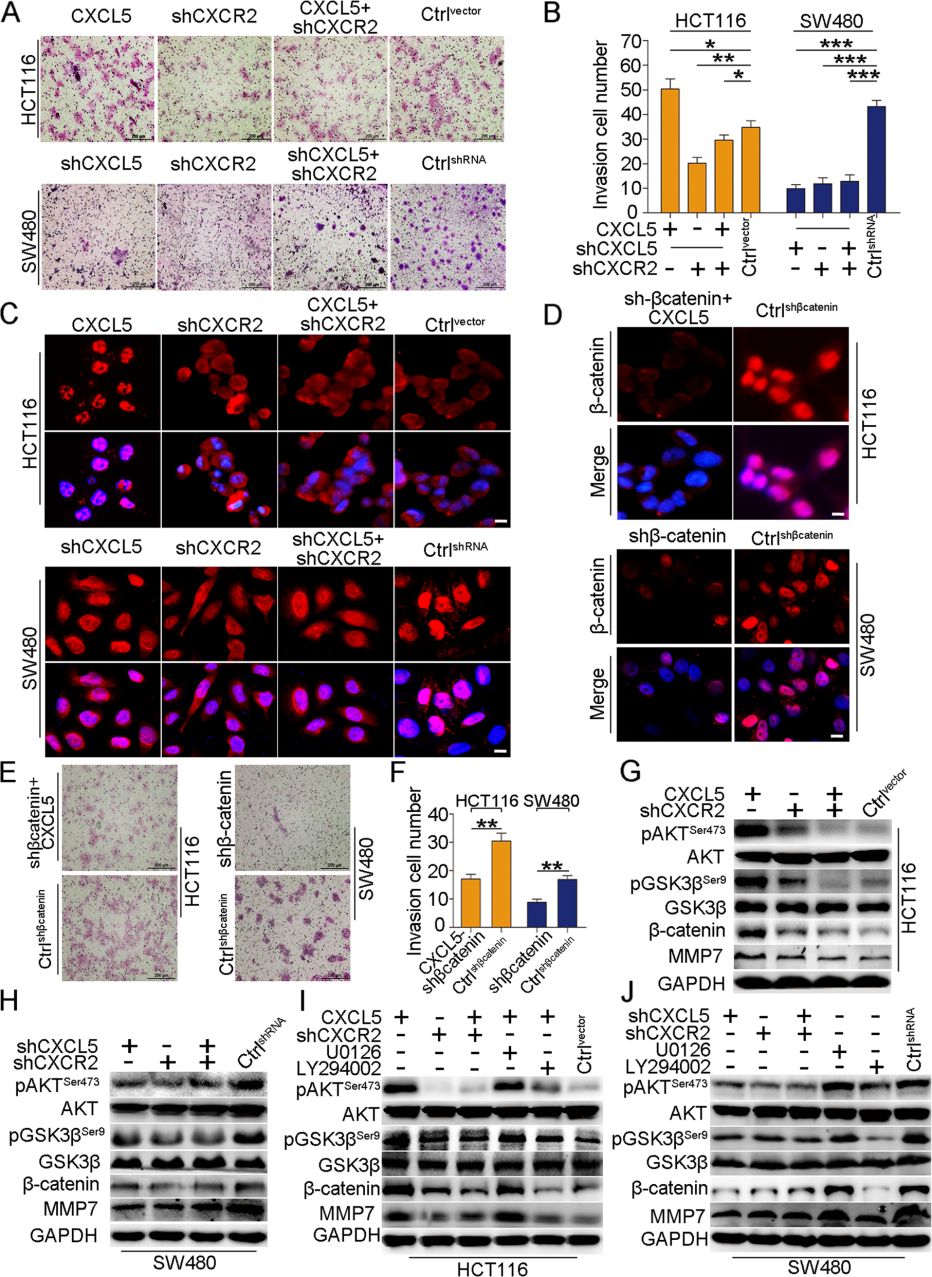
The CXCL5/CXCR2 axis regulates CRC cell invasion through the AKT/GSK3β/β-catenin/MMP7 pathway. **a** & **b** CXCL5 promotes CRC cell invasion in a CXCR2-dependent manner in HCT116. Inhibition of CXCL5 reverses this process in a CXCR2-dependent manner in SW480. Scale, 200 μm. Data are presented as the mean ± SD. **c** Immunofluorescence images show that CXCL5 promotes translocation of β-catenin in a CXCR2-dependent manner in HCT116. Inhibition of CXCL5 reverses this process in a CXCR2-dependent manner in SW480. Scale, 50 μm. **d** The inhibitory effects of β-catenin- shRNA on β-catenin in HCT116^CXCL5^ and SW480 cells. Scale, 50 μm. **e** & **f** Inhibition of β-catenin decreases the invasion of CRC cells. Scale, 200 μm. Data are presented as the mean ± SD. **g** & **h** CXCL5/CXCR2 enhances the expression of MMP7 through activation of the AKT/GSK3β/β-catenin pathway. **i** & **j** Inhibition of the AKT pathway (LY294002) instead of the ERK pathway (U0126) reverses the activation of GSK3β/β-catenin and upregulates the expression of MMP7. *P < 0.05, **P < 0.01, ***P < 0.001

We next assessed the contribution of the CXCL5/CXCR2 axis to CRC cell invasion. We found that ectopic expression of CXCL5 was able to promote cell invasion, and the difference in the number of invading cells was statistically significant compared with the control group in HCT116. The knock-down of CXCR2 also reduced the number of invading cells. Consistently, inhibition of CXCL5 or (and) CXCR2 decreases invading cell number in SW480 group (Fig. 6a and b). Several studies have demonstrated that β-catenin is able to translocate to the tumor cell nucleus and function as an oncogene to promote CRC cell invasion [18]. Consistently, our results revealed that CXCL5 enhanced the translocation of β-catenin to the nucleus in HCT116^CXCL5^ cells. The inhibition of CXCR2 led to an accumulation of β-catenin in the cytoplasm instead of translocation to the nucleus. However, β-catenin accumulated primarily in the nucleus of the SW480^shRNA^ cells, and CXCL5 and/or CXCR2 inhibition resulted in a cytoplasmic distribution of β-catenin (Fig. 6c). After down-regulating the expression of β-catenin in HCT116^CXCL5^ and SW480^shRNA^ cells (Fig. 6d), we found the inhibition of β-catenin eliminated the CXCL5-induced invasion in HCT116^CXCL5^ and SW480 cells, as the number of invading cells was significantly lower than in the control group (*P* < 0.01) (Fig. 6e and f). These data indicate that CXCL5/CXCR2 may promote CRC cell invasion through the regulation of β-catenin, as demonstrated by the redistribution of cytosolic and nuclear β-catenin.

As discussed above, the CXCL5/CXCR2 axis is able to regulate both the ERK and AKT pathways. However, the AKT pathway has minimal effects on CRC cell migration. Thus, we hypothesized that the AKT pathway may be involved in the regulation of CRC cell invasion. Immunoblot results revealed that the upregulation of pAKT^Ser473^ in HCT116^CXCL5^ enhanced the expression of β-catenin and the downstream factor pGSK3β^Ser9^. Knock-down of CXCR2 in HCT116 and HCT116^CXCL5^ cells blocked this process (Fig. 6g). Downregulation of CXCL5 and/or CXCR2 in SW480 cells inhibited the phosphorylation of AKT^Ser473^, the expression of β-catenin and pGSK3β^Ser9^ (Fig. 6h). These results indicate that CXCL5-enhanced phosphorylation of AKT^Ser473^ may be able to increase β-catenin levels in CRC cells in a CXCR2-dependent manner.

As previously reported, MMP7 may be one of the targets of β-catenin in CRC [19]. Consequently, we examined MMP7 expression in CRC cells. Our results demonstrate that the regulation of MMP7 coincides with alterations in β-catenin levels (Fig. 6g and h). Moreover, we found inhibition of pAKT^Ser473^ led to decrease of MMP7. However, the inhibition of the ERK pathway by U0126 has no effect on this process (Fig. 6i and j). Taken together, these data indicate that CXCL5 is able to promote CRC cell invasion in a CXCR2-dependent manner. This process may involve regulation of the AKT/GSK3β/β-catenin/MMP7 pathway.

### CXCL5/CXCR2 increases CRC liver metastasis in vivo

**Fig. 7:**
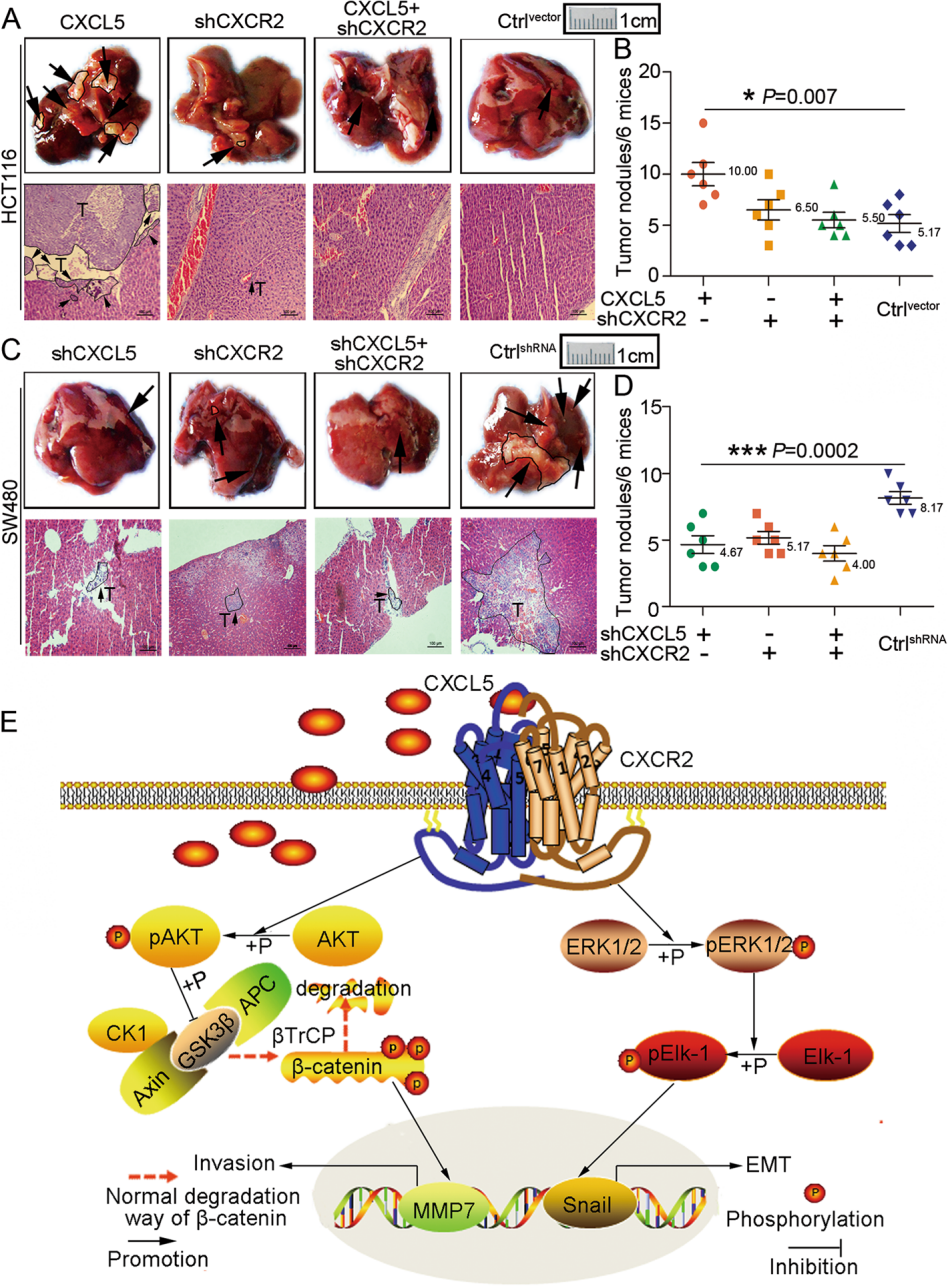
CXCL5/CXCR2 promotes CRC liver metastasis in vivo and schematic diagram. **a** & **c** Representative images of liver metastasis in the indicated HCT116 and SW480 cells in vivo. Scale, 100 μm. Six mice are included in each group. **b** & **d** The number of metastatic nodules in each indicated group. Data are presented as the mean ± SD. *P < 0.05, ***P < 0.001. **e** Schematic diagram shows that tumor-derived CXCL5 promotes CRC cell migration by inducing EMT through the activation of ERK/Elk-1/Snail and enhances invasion through the activation of AKT/GSK3β signaling, which inhibits the degradation of β-catenin. Accumulated β-catenin in the cytoplasm translocates to the nucleus to promote the expression of MMP7, which facilitates cell invasion

To determine whether the CXCL5/CXCR2 axis is involved in CRC metastasis in vivo, we generated a liver metastasis model using nude mice. Results revealed that more metastatic nodules were observed in the HCT116^CXCL5^ group, and fewer metastatic lesions were observed in the HCT116^shCXCR2^ and HCT116^CXCL5-shCXCR2^ groups compared with the control group. The difference in the number of nodules was statistically significant (Fig. 7a and b). However, the SW480^shCXCL5^, SW480^shCXCR2^, and SW480^shCXCL5-shCXCR2^ groups formed fewer metastatic nodules compared with the control group. The number of nodules in each group was significantly less than the control group (Fig. 7c and d). These indicate that CXCL5/CXCR2 axis is able to promote CRC liver metastasis in vivo. A schematic diagram of our study is shown in Fig. 7e.

## Discussion

Chemokines link the extra-tumoral microenvironment to the tumor and facilitate the progression of CRC [20]. For this reason, we examined the expression of several chemokines in CRC and preliminarily verified the upregulation of CXCL5 in CRC. Previous studies have reported that CXCL5 produced by fibroblasts plays a prominent role in the progression, growth and spread of various types of cancer [15, 21]. However, in our study, we revealed that CRC cells, rather than fibroblasts, are able to secrete CXCL5. This promotes liver metastasis in vivo and contributes to CRC cell migration via the ERK pathway through the induction of EMT, as well as invasion via the AKT pathway in vitro.

As described above, we first screened the expression profiles of several chemokines. The functions of the most upregulated chemokines have been studied in various cancers, including CRC. We primarily concentrated on CXCL5 in our study. Next, we employed two cohorts of clinical samples to determine the levels of CXCL5 expression. Our results demonstrated that the expression of CXCL5 was clearly up-regulated in CRC tissues. In the second cohort, we linked the results to clinical follow-up data. CXCL5 was significantly correlated with tumor size, Dukes’ stage, tumor invasion, lymph node localization, and liver metastasis. The upregulation of CXCL5 in tumor tissues was also positively correlated with a poor prognosis in CRC patients. CXCL5^high^ patients had lower OS and DFS rates compared with CXCL5^low^ patients. In contrast, Frank et al. demonstrated that the absence of CXCL5 expression in tumor tissues was correlated with poor prognosis in CRC, and they also found that CXCL5 was overexpressed in tumor tissues [22]. They attributed these phenomena to intratumoral infiltration of T cells and neutrophils. However, according to our unpublished data, the neutrophils infiltrating the tumor region are more likely to be N2-type protumoral neutrophils, rather than classical N1-type antitumoral neutrophils. We suspect that the reason for this conflict may be that there were too many stage I and stage II patients (67.1% vs 61.5% in our study) and no stage IV patients, even though there was no significant difference in the cohort distribution. Perhaps the protumoral function of T cell infiltration is dominant only in the early stages of cancer progression [23]. As the disease progresses, the influence of CXCL5 on tumor cells becomes the dominant cause of cancer. Furthermore, our results revealed that CXCL5 is an independent prognostic factor for DFS, but not for OS. This may be because not all of the patients died from cancer recurrence in the OS group. Some patients may have died from cardiovascular disease. Taken together, these data indicate that CXCL5 is highly expressed in CRC tissues and predicts a poor prognosis for CRC patients.

We also observed that CXCL5 was primarily expressed in tumor lesions, rather than the mesenchyme of cancer tissue, and CRC cell lines were capable of secreting CXCL5. This result indicates that the effect of CXCL5 may likely depend on an autocrine signaling pathway in CRC, rather than being fibroblast-dependent as reported in other types of cancer [15]. To further understand this process, we constructed CRC cell lines that over-expressed or under-expressed CXCL5 and employed in vivo nude mouse models to elucidate the effects of CXCL5 on CRC liver metastasis. Upregulated expression of CXCL5 increased CRC-derived liver metastasis in the nude mouse model, which was dependent on CXCR2. In line with these results, in vitro experiments also showed that ectopic expression of CXCL5 promoted CRC cell migration and invasion.

EMT is characterized as a dynamic and reversible biological behavior [19, 24]. When cancer cells escape from the primary site to metastasis, subsets of cancer cells undergo a morphological conversion from an epithelial-like phenotype to a mesenchyme-like phenotype [25]. This process is characterized by the loss of epithelial cell junction proteins, such as E-cadherin, and an upregulation of mesenchymal markers, such as Vimentin and N-cadherin [26]. In this study, we found that CRC cells with high levels of CXCL5 expressed low levels of E-cadherin and ZO-1 and high levels of N-cadherin and Vimentin. The inhibition of CXCR2 was able to reverse this process. CXCR2 was initially recognized as a G protein-coupled transmembrane chemokine receptor involved in a variety of pathways, such as the ERK, PI3K/AKT, JNK and STAT3 pathways [27]. In our study, we verified that both the ERK and AKT pathways can be activated by CXCL5/CXCR2. We next investigated that inhibition of the ERK pathway instead of AKT pathway significantly leads to the alteration of EMT markers. These results indicate that CXCL5/CXCR2 may induce EMT through the ERK pathway. It has been reported that ERKs can activate several downstream transcription factors through phosphorylation to control the expression of specific genes [28]. Elk-1 is one of those downstream transcription factors [29]. In our study, we confirmed that inhibition of the ERK pathway leads to the downregulation of pElk-1, which is accompanied by the suppression of Snail.

Snail is an important transcription factor that acts as a repressor of E-cadherin expression and an inducer of EMT in various types of cancer [30]. Here, we show that Snail is upregulated upon activation of the CXCL5/CXCR2 axis. Furthermore, the knock-down of Snail can reverse CXCL5/CXCR2-induced EMT in CRC cells. Additionally, inhibition of the ERK pathway, rather than the AKT pathway, eliminates the effects of the CXCL5/CXCR2 signaling axis on Snail. These data indicate that CXCL5/CXCR2 induces EMT via the ERK/Elk-1/Snail pathway in CRC cells.

Another important finding of our study was that the activation of the AKT pathway by the CXCL5/CXCR2 axis may be involved in promoting CRC cell invasion. We found that the CXCL5/CXCR2 axis was capable of promoting CRC cell invasion, but the mechanisms behind this were unclear. MMPs are overexpressed in many cancers and are capable of mediating invasion and metastasis via ECM degradation [31]. It has been reported that MMP7 is a critical factor in the regulation of CRC cell invasion [32]. In our investigation, we revealed that CXCL5/CXCR2 was responsible for the potentiation of MMP7 expression and the inhibition of the AKT pathway, and the inhibitor LY294002 instead of U0126 interfered with this process. GSK3β is a downstream effector of AKT, and pAKT can suppress the function of GSK3β by phosphorylating its serine residue [24]. GSK3β is a key component of the destruction complex that facilitates the phosphorylation of β-catenin in CRC [33]. Phosphorylated β-catenin can be recognized by β-TrCP, which ubiquitinates pβ-catenin, and the ubiquitinated pβ-catenin is then degraded by proteasomes [34]. Here, we report that activation of the AKT pathway enhances the phosphorylation of downstream GSK3β and the expression of total β-catenin in CRC cells, perhaps because the phosphorylation of GSK3β results in the separation of the destruction complex and stabilization of total β-catenin. Additionally, we also found that the CXCL5/CXCR2 axis is capable of enhancing the translocation of β-catenin from the cytoplasm to the nucleus where β-catenin can bind to several promoters, including MMP7. Moreover, knock-down of β-catenin in CRC cells decreases the number of invading cells. Altogether, these results demonstrate that the CXCL5/CXCR2 axis promotes CRC cell invasion through the AKT/GSK3β/β-catenin/MMP7 pathway.

Although the function of CXCL5/CXCR2 on tumor metastasis is confirmed in vivo. however, it is difficult to translate the data acquired in vivo to human totally. Because there are at least two chemokines, CXCL5 and CXCL6, which are able to bind to CXCR2 in human [35]. While CXCL5 and CXCL6, also called LIX or GCP-2, are the same chemokine in mice. Human CXCL5 and CXCL6 are homologically related to CXCL5 in mice [36]. In addition, mice don’t have CXCL8 but CXCL8 and human CXCL6 are the only ligands to bind to both CXCR1 and CXCR2 in human [37, 38]. Thus upregulating CXCL5 expression in mice may have a more drastic effect than upregulation of only one of the three chemokines in human.

## Conclusion

In conclusion, our results indicate that CXCL5 is over-expressed in CRC, which is predictive of a poor prognosis for CRC patients. We also demonstrate a novel role for CXCL5 in the induction of EMT and promotion of CRC cell migration through the ERK/Elk-1/Snail pathway in a CXCR2-dependent manner. Furthermore, we reveal that CXCL5/CXCR2 can potentiate CRC cell invasion via the AKT/GSK3β/β-catenin/MMP7 pathway rather than the ERK pathway. Thus, inhibition of the CXCL5/CXCR2 signaling pathway may be a promising target for therapies for CRC patients.

## Methods

### Chemokine microarray analysis

The profiles of chemokines were examined using a Human Chemokine Antibody Array C1 kit (RayBiotech, USA). This experiment was performed according to the manufacturer’s instructions and supported by the BioTNT Corporation, Shanghai. The levels of certain chemokines in the tumor tissues were more than 2.0-fold higher, which we defined as “significant upregulation”. We also defined a 1.5- to 2.0-fold change as “insignificant upregulation”, a 0.67- to 1.5-fold change as “no difference”, a 0.5- to 0.67-fold change as “nonsignificant downregulation”, and a less than 0.5 change as “significant downregulation”.

### Patients and follow-up

Two independent cohorts of colorectal cancer patients are enrolled in our study. All these patients were diagnosed specifically by pathology as CRC (before or after surgery) and treated with laparoscopic surgery in Minimally Invasive Surgery Centre, Ruijin Hospital, Shanghai Jiaotong University. Ethics approval for use of human specimen was obtained from the Biomedical Ethics Committee of Ruijin Hospital. All the tumor tissues and paired peritumoral normal tissues contained in Cohort 1 were collected from patients undergoing operation between Nov.2015 and Mar.2016. Tissues of Cohort 2 were collected from patients undergoing operation from 2010 to 2011. Clinical and pathological data were collected. All the patients who had received preoperative treatment such as radiation or chemotherapy were excluded. Pathological staging of CRC tumor was performed in accordance to the TNM classification [39]. The follow-up data were acquired at 2-month intervals through outpatient visits, telephone calls, or office visits. The follow-up data were ceased in August 2015.

### Cell lines

The human CRC cell lines used in our study were purchased from the American Type Culture Collection (ATCC, USA) and preserved in liquid nitrogen by the Shanghai Digestive Surgery Institute and routinely tested for mycoplasma contamination using PCR. These cell lines were authenticated prior to being used in our study. The SW480 cell line was cultured using Leibovitz’s L-15 medium, and the HCT116 cell line was cultured using McCoy’s 5A medium with 10% FBS, penicillin (10^7^ U/L), and streptomycin (10 mg/L) at 37 °C with 5% CO_2_ in an incubator.

### Liver metastasis in in vivo nude mouse models

Animal experiments were performed in accordance with the ethical guidelines issued by the Ethics Committee of Shanghai Jiaotong University. Male BALB/c nu/nu mice (4 weeks old) were purchased from the Chinese Academy of Sciences, Shanghai and raised in a specific-pathogen-free environment. For the construction of the liver metastasis model, 6 nude mice were included in each group (HCT116^CXCL5^, HCT116^shCXCR2^, HCT116^CXCL5-shCXCR2^ and HCT116^vector^ groups as well as SW480^shCXCL5^, SW480^shCXCR2^, and SW480^shCXCL5-shCXCR2^, and SW480^shRNA^ groups were injected into the spleens of the mice). After being anesthetized with an intraperitoneal injection of 1% pentobarbital sodium (50 mg/kg), abdominal surgeries were performed to expose the spleen and slowly inject it with 1×10^7^ cells suspended in 150 μL PBS. The spleen was then returned to the abdominal cavity, and the abdomen was closed. The cells were then transported by the circulatory system to the liver to spontaneously form metastatic lesions. The mice were euthanized by cervical decapitation 6 weeks after injection to examine the liver metastases of tumor cells. Liver metastases were confirmed by HE staining.

### Statistical analysis

All of the statistical analysis were performed using SAS 8.0, SPSS 16.0 or R software 3.1.2 (R Core Team). The Pearson *χ* ^2^ test and Fisher’s exact probability method were used to analyze the relationship between CXCL5 and clinical features. Overall Survival and Disease Free Survival curves were plotted using the Kaplan-Meier method, and differences between the two groups were determined using a log-rank test. Univariate and multivariate analyses were performed using the Cox regression model. A nomogram was formulated based on the results of the multivariate analysis and plotted using R software. All experiments were performed in triplicate. *P* < 0.05 was considered to be statistically significant.

## Additional files

Additional file 1: Figure S1. X-tile analysis of survival data in CRC patients reveals a continuous distribution based on CXCL5 staining score. The plot shows the *χ*2 log-rank values produced when dividing the cohort with one cut-point, producing high, and low subsets. The X-axis represents all potential cut-points from low to high (left to right) that defines a low subset, whereas the Y-axis represents cut-points from high to low (top to bottom), that defines a high subset. Red coloration of cut-point indicates an inverse correlation with survival, whereas green coloration represents direct associations (A). The optimal cut-point occurs at the brightest pixel (red). The cut-point highlighted by the white circle in A is shown on a histogram of the entire cohort (B), and a Kaplan-Meier plot (C, low subset grey, high subset light green). (TIF 4341 kb)

Additional file 2: Figure S2. Increased expression of CXCL5 in CRC tissues and calibration curve. A: An immunoblot showing that CXCL5 is overexpressed in cancer tissues compared with the peritumoral normal tissues. B: Calibration plots for predicting DFS 3 years after surgery. C: Calibration plots for predicting DFS 5 years after surgery. (TIF 2163 kb)

Additional file 3: Tables and Methods. **Table S1**, shRNA Sequences; **Table S2**, antibodies for immunoblot; **Table S3**, antibodies for immunofluorescence; **Table S4**, correlations between CXCL5 expression and clinical characteristics in CRC patients; **Table S5**, univariate and multivariate analyses of CXCL5 expression in CRC patients; **Table S6**, pathological stages of patients in Fig. 2A&B; Supplementary methods. (DOC 178 kb)

Additional file 4: Figure S3. The expression of CXCL5 in CRC cell lines and CXCL5/CXCR2 promotes CRC cell migration through the induction of EMT. A: ELISAs examining CXCL5 expression levels in CRC cell lines supernatants. B: Immunofluorescence analysis verifying the effects of upregulation of CXCL5 using a CXCL5-vector and shRNA-inhibition of CXCL5 and CXCR2. Magnification, 400×. C: Morphological changes in the indicated cells. Magnification, 200×. Scale, 200 μm. (TIF 16430 kb)

Additional file 5: Figure S4. CXCL5/CXCR2 induces EMT in CRC cells via the ERK/Elk-1 pathway. A & B: Morphological changes after treatment with LY294002 or U0126 in HCT116 and SW480 cells. Scale, 200 μm. C & E: Representative images of the transwell migration assays after treatment with LY294002 or U0126 in HCT116 and SW480 cells. Scale, 200 μm. Treatment with U0126 decreases the migrated cell number. D & F: The column graph displays the migrated cell number of the indicated cells. Data are presented as the mean ± SD. *P < 0.05, **P < 0.01, ***P < 0.001. (TIF 12172 kb)

## Abbreviations

BCA: Bicinchoninic acid
CRC: Colorectal carcinoma
EMT: Epithelial mesenchymal transition
GFP: Green fluorescence protein
shRNA: Short hairpin RNA
