# Supplementary figures and images for: Tumor-derived CXCL5 promotes human colorectal cancer metastasis through activation of the ERK/Elk-1/Snail and AKT/GSK3β/β-catenin pathways
Source: Mol Cancer. 2017 Mar 29;16:70. doi: 10.1186/s12943-017-0629-4 (PMC5372323; doi:10.1186/s12943-017-0629-4)

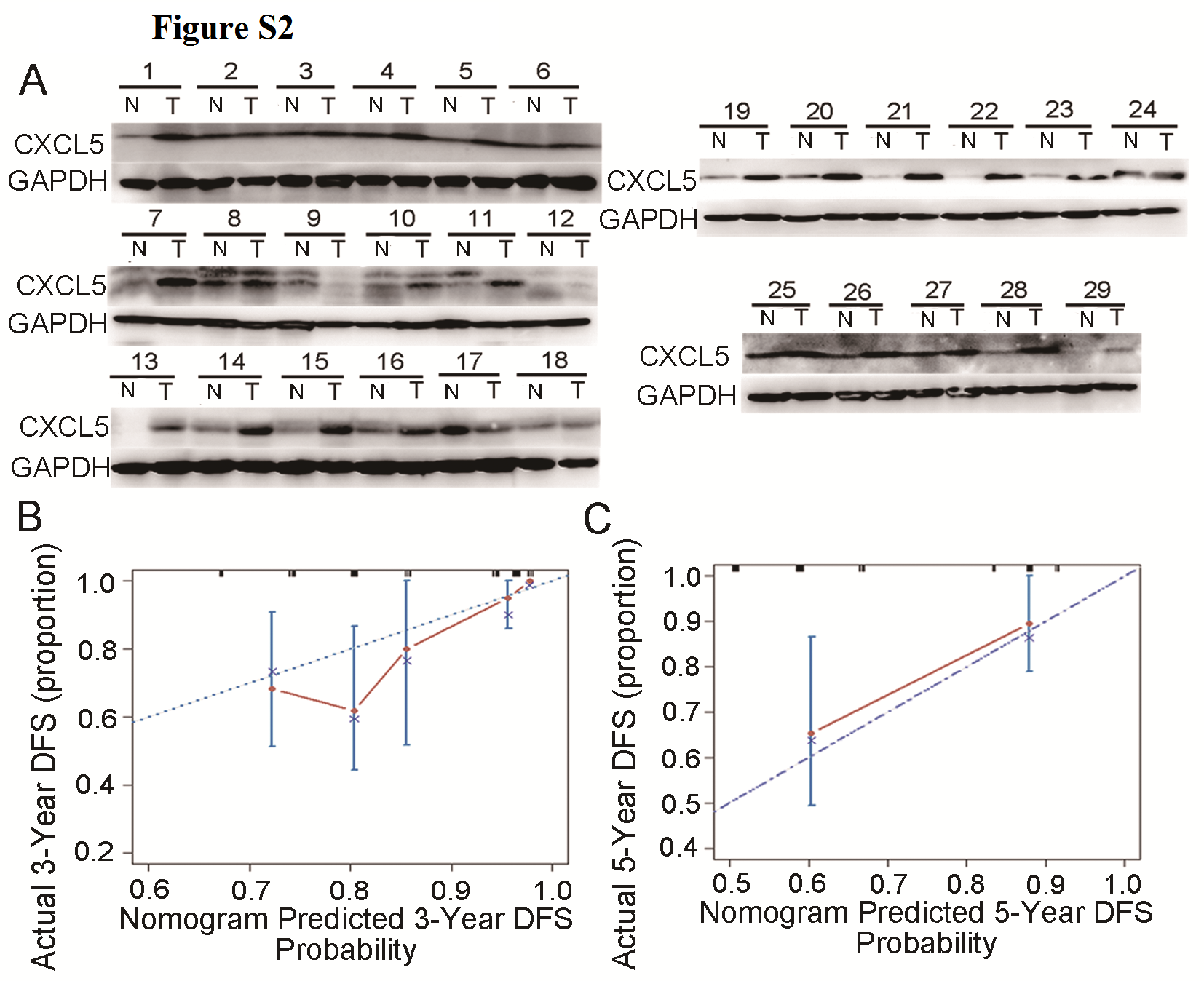

Supplement: Supplementary file 1 — X-tile analysis of survival data in CRC patients reveals a continuous distribution based on CXCL5 staining score. The plot shows the χ2 log-rank values produced when dividing the cohort with one cut-point, producing high, and low subsets. The X-axis represents all potential cut-points from low to high (left to right) that defines a low subset, whereas the Y-axis represents cut-points from high to low (top to bottom), that defines a high subset. Red coloration of cut-point indicates an inverse correlation with survival, whereas green coloration represents direct associations (A). The optimal cut-point occurs at the brightest pixel (red). The cut-point highlighted by the white circle in A is shown on a histogram of the entire cohort (B), and a Kaplan-Meier plot (C, low subset grey, high subset light green). (TIF 4341 kb) [file 12943_2017_629_MOESM1_ESM.tif]

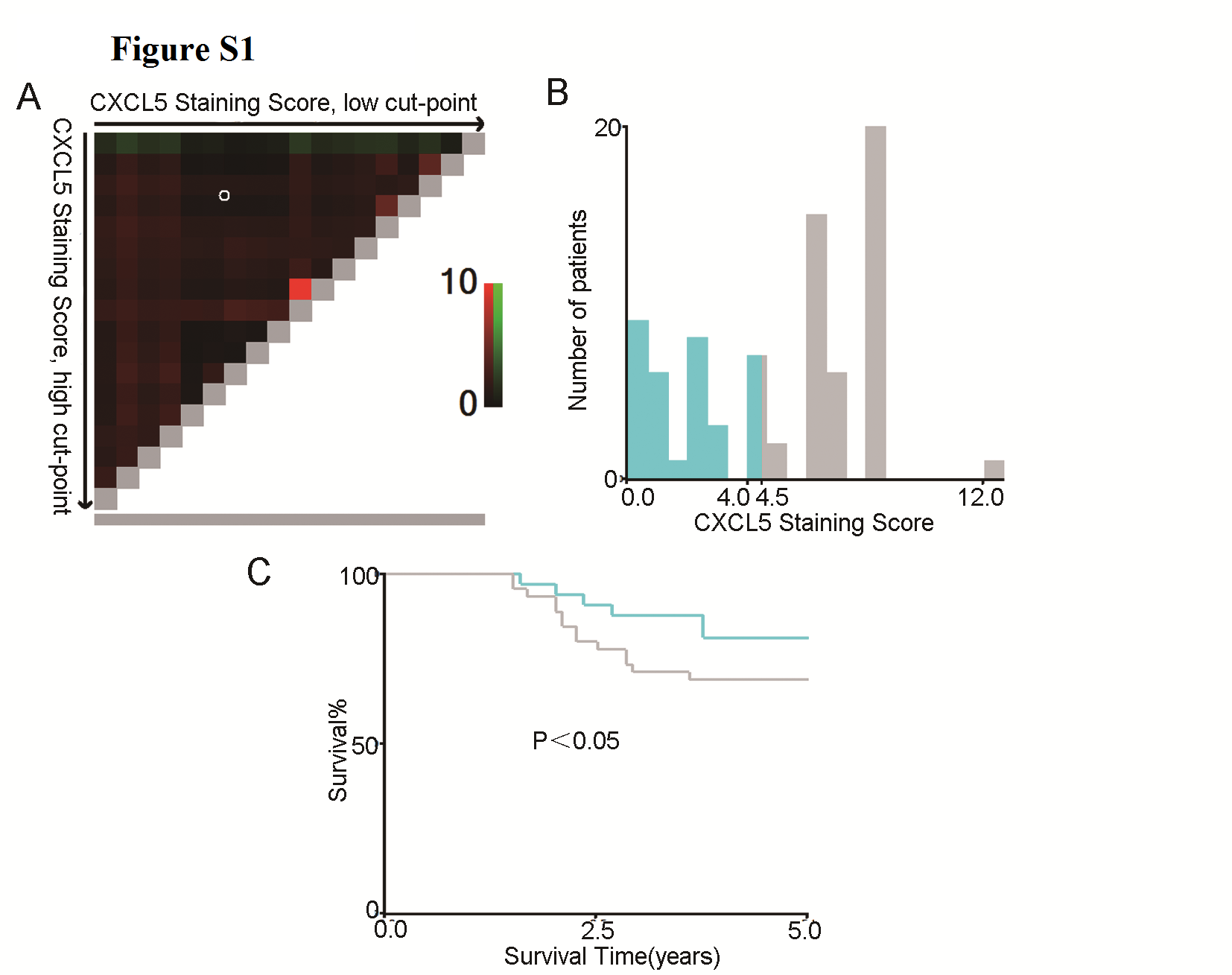

Supplement: Supplementary file 2 — Increased expression of CXCL5 in CRC tissues and calibration curve. A: An immunoblot showing that CXCL5 is overexpressed in cancer tissues compared with the peritumoral normal tissues. B: Calibration plots for predicting DFS 3 years after surgery. C: Calibration plots for predicting DFS 5 years after surgery. (TIF 2163 kb) [file 12943_2017_629_MOESM2_ESM.tif]

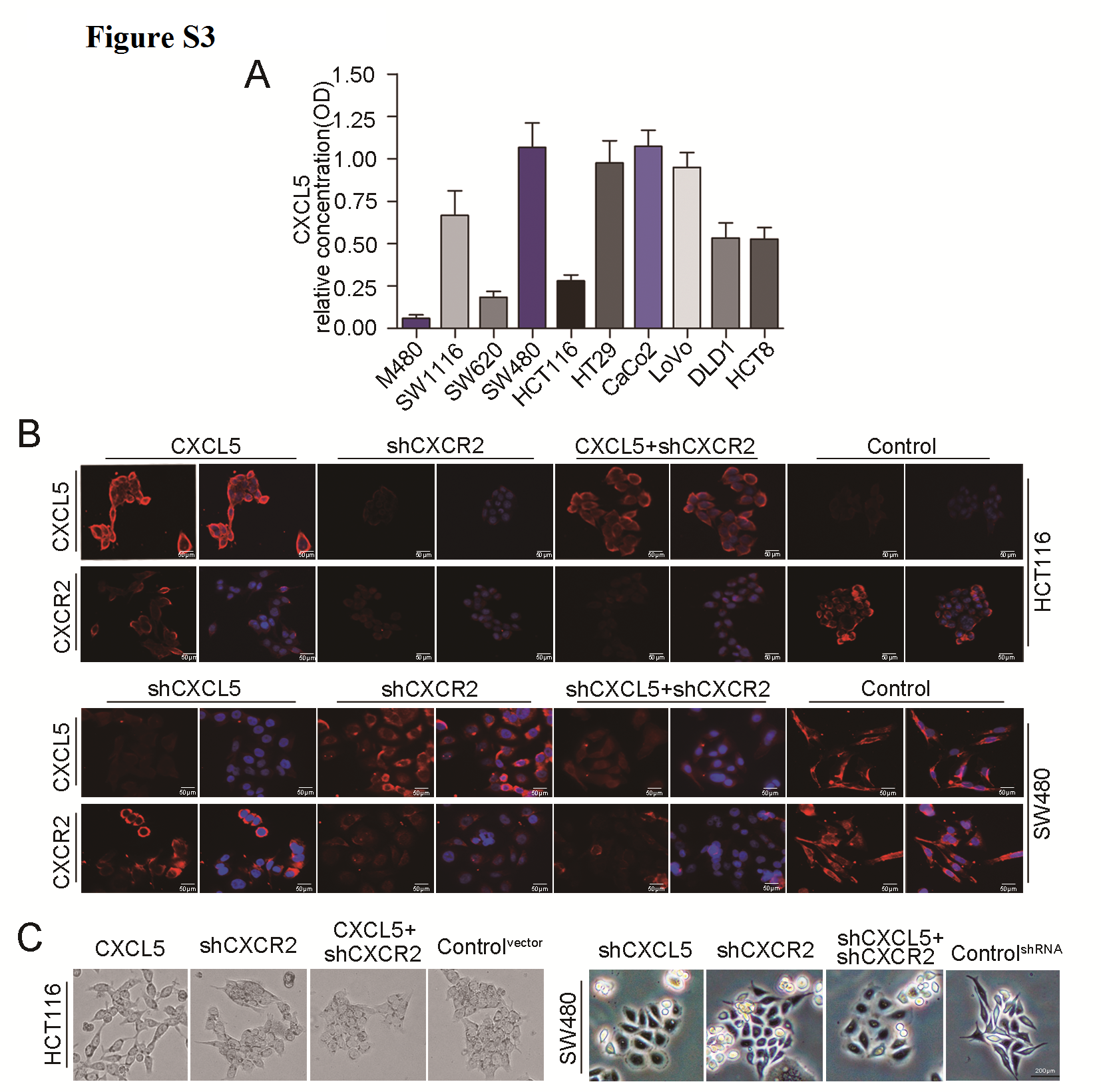

Supplement: Supplementary file 4 — The expression of CXCL5 in CRC cell lines and CXCL5/CXCR2 promotes CRC cell migration through the induction of EMT. A: ELISAs examining CXCL5 expression levels in CRC cell lines supernatants. B: Immunofluorescence analysis verifying the effects of upregulation of CXCL5 using a CXCL5-vector and shRNA-inhibition of CXCL5 and CXCR2. Magnification, 400×. C: Morphological changes in the indicated cells. Magnification, 200×. Scale, 200 μm. (TIF 16430 kb) [file 12943_2017_629_MOESM4_ESM.tif]

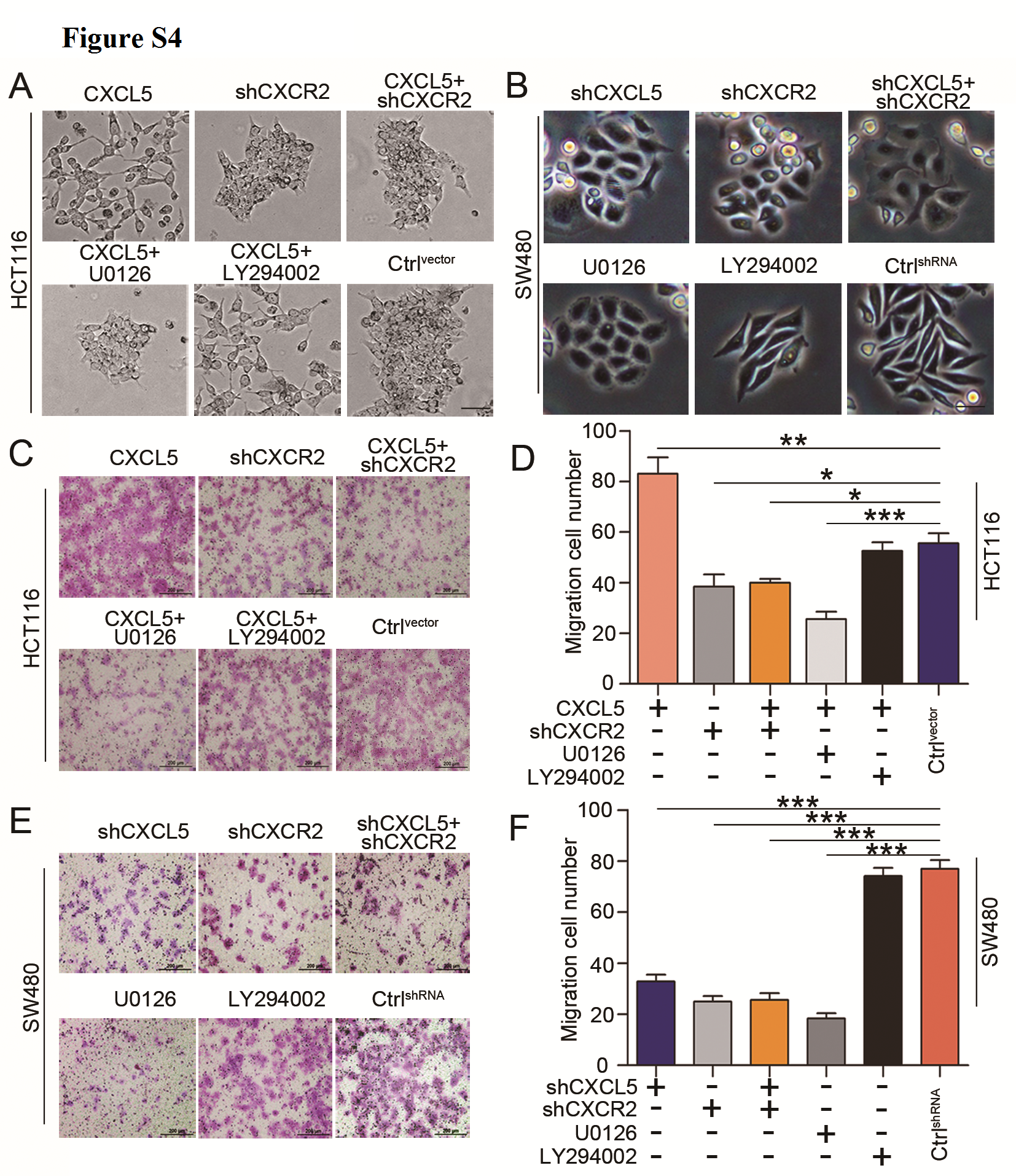

Supplement: Supplementary file 5 — CXCL5/CXCR2 induces EMT in CRC cells via the ERK/Elk-1 pathway. A & B: Morphological changes after treatment with LY294002 or U0126 in HCT116 and SW480 cells. Scale, 200 μm. C & E: Representative images of the transwell migration assays after treatment with LY294002 or U0126 in HCT116 and SW480 cells. Scale, 200 μm. Treatment with U0126 decreases the migrated cell number. D & F: The column graph displays the migrated cell number of the indicated cells. Data are presented as the mean ± SD. *P < 0.05, **P < 0.01, ***P < 0.001. (TIF 12172 kb) [file 12943_2017_629_MOESM5_ESM.tif]
