# Supplementary material for: Tumor-derived CXCL5 promotes human colorectal cancer metastasis through activation of the ERK/Elk-1/Snail and AKT/GSK3β/β-catenin pathways
Source: Mol Cancer. 2017 Mar 29;16:70. doi: 10.1186/s12943-017-0629-4 (PMC5372323; doi:10.1186/s12943-017-0629-4)
Supplement: Supplementary file 3 — Table S1, shRNA Sequences; Table S2, antibodies for immunoblot; Table S3, antibodies for immunofluorescence; Table S4, correlations between CXCL5 expression and clinical characteristics in CRC patients; Table S5, univariate and multivariate analyses of CXCL5 expression in CRC patients; Table S6, pathological stages of patients in Fig. 2A&B; Supplementary methods. (DOC 178 kb) [file 12943_2017_629_MOESM3_ESM.doc]

**Table S1 shRNA Sequences**

| shRNA | Stem Sequence |
| --- | --- |
| **CXCL5-LV3-shRNA#1**  CXCL5-LV3-shRNA#2  CXCL5-LV3-shRNA#3 | **GATCAGTAATCTGCAAGTGTT**  CGGGAAGGAAATTTGTCTTGA  TGAATTGTAGGTGACTATTAT |
| CXCR2-LV3-shRNA#1  **CXCR2-LV3-shRNA#2**  CXCR2-LV3-shRNA#3 | CTCATTAGGATGGCTAGTATC  **CCGTCTACTCATCCAATGTTA**  TCCTCAAGATTCTAGCTATAC |
| **Snail-shRNA#1**  Snail-shRNA#2  Snail-shRNA#3 | **CCACTCAGATGTCAAGAAGTA**  CCACTCAGATGTCAAGAAGTA  GCAGGACTCTAATCCAGAGTT |
| β-catenin-shRNA#1  **β-catenin-shRNA#2**  β-catenin-shRNA#3 | GCTCCTGTCTAATGCTTAGTT  **TTGTTATCAGAGGACTAAATA**  CGGATTAAGTTTCCAGACAAT |

***Bold fonts represent the most effective sequence**

**Table S2 Antibodies for immunoblot**

| **Antibody(Source)** | **Dilution for Immunoblot** | **Manufacturer** |
| --- | --- | --- |
| CXCL5(Mouse) | 1:500 | R&D, USA |
| CXCR2(Rabbit) | 1:500 | Abcam, UK |
| E-cadherin(Rabbit) | 1:1000 | CST, USA |
| ZO-1(Rabbit) | 1:1000 | CST, USA |
| N-cadherin(Rabbit) | 1:1000 | CST, USA |
| Vimentin(Rabbit) | 1:1000 | CST, USA |
| Snail(Rabbit) | 1:1000 | CST, USA |
| Slug(Rabbit) | 1:1000 | CST, USA |
| pSTAT3^Tyr705^(Rabbit) | 1:1000 | CST, USA |
| STAT3(Rabbit) | 1:1000 | CST, USA |
| pJNK^Thr183/Tyr185^(Rabbit) | 1:1000 | CST, USA |
| JNK(Rabbit) | 1:1000 | CST, USA |
| pERK1/2^Thr202/Tyr204^(Rabbit) | 1:1000 | CST, USA |
| ERK(Rabbit) | 1:1000 | CST, USA |
| pElk-1^Ser383^(Rabbit) | 1:1000 | CST, USA |
| Elk-1(Rabbit) | 1:1000 | CST, USA |
| pAKT^Ser473^(Rabbit) | 1:1000 | CST, USA |
| AKT(Rabbit) | 1:1000 | CST, USA |
| pGSK3β^Ser9^(Rabbit) | 1:1000 | CST, USA |
| GSK3β(Rabbit) | 1:5000 | Abcam, UK |
| β-catenin(Rabbit) | 1:1000 | Abclonal, China |
| αSMA | 1:500 | Abcam, UK |
| GAPDH(Mouse) | 1:1000 | CST, USA |

**Table S3 Antibodies for immunofluorescence**

| **Antibody(Source)** | **Dilution for Immunoblot** | **Manufacturer** |
| --- | --- | --- |
| CXCL5(Mouse) | 1:100 | R&D, USA |
| CXCR2(Rabbit) | 1:100 | Abcam, UK |
| E-cadherin(Rabbit) | 1:100 | Abcam, UK |
| Vimentin(Rabbit) | 1:100 | CST, USA |
| β-catenin(Rabbit) | 1:50 | Abclonal, China |
| αSMA | 1:100 | Abcam, UK |

**Table S4** Correlations between CXCL5 expression and clinical characteristics in CRC patients

| Clinicopathologic Parameters | CXCL5 expression | | Statistical  Value | *P*  Value |
| --- | --- | --- | --- | --- |
|  | High | Low |  |  |
| Total  Gender | 48 | 30 |  |  |
| Male | 23 | 18 | 1.086 | 0.297 |
| Female | 25 | 12 |  |  |
| Age |  |  |  |  |
| ＜65 | 28 | 19 | 0.194 | 0.660 |
| ≥65 | 20 | 11 |  |  |
| Histology |  |  |  |  |
| Tubular | 38 | 28 | 3.534 | 0.171 |
| Mucinous | 9 | 2 |  |  |
| Papillary | 1 | 0 |  |  |
| Tumor location |  |  |  |  |
| Right hemicolon | 16 | 9 | 3.593 | 0.309 |
| Transverse colon | 0 | 1 |  |  |
| Left hemicolon | 5 | 1 |  |  |
| Sigmoid+Rectum | 26 | 19 |  |  |
| Tumor size |  |  |  |  |
| ≥5cm | 27 | 10 | 3.945 | **0.047** |
| ＜5cm | 21 | 20 |  |  |
| Dukes stage |  |  |  |  |
| Ⅰ | 5 | 10 | 2.735 | **0.003** |
| Ⅱ | 21 | 12 |  |  |
| Ⅲ | 16 | 8 |  |  |
| Ⅳ | 6 | 0 |  |  |
| TNM stage |  |  |  |  |
| T_1_ | 3 | 5 | 2.372 | **0.009** |
| T_2_ | 3 | 4 |  |  |
| T_3_ | 8 | 8 |  |  |
| T_4_ | 34 | 13 |  |  |
| N_0_ | 20 | 22 | 2.927 | **0.002** |
| N_1_ | 14 | 6 |  |  |
| N_2_ | 14 | 2 |  |  |
| M_0_ | 42 | 30 | 2.016 | **0.022** |
| M_1_ | 6 | 0 |  |  |
| CEA |  |  |  |  |
| ≥5 ng/mL | 25 | 11 | 1.781 | 0.182 |
| ＜5 ng/mL | 23 | 19 |  |  |

**Table S5** Univariate and multivariate analyses of CXCL5 expression in CRC patients

| Parameters | **OS** | | | **DFS** | | |
| --- | --- | --- | --- | --- | --- | --- |
|  | HR | 95% CI | *P* | HR | 95% CI | *P* |
| **Univariate analysis** |  |  |  |  |  |  |
| Age (＜65vs ≥65,year) | 1.003 | 0.410-2.454 | 0.995 | 0.794 | 0.355-1.773 | 0.573 |
| Gender(female vs male) | 1.812 | 0.740-4.433 | 0.193 | 2.190 | 0.955-5.024 | 0.064 |
| Histology |  |  |  |  |  |  |
| Papillary | 1.000 | .. | 0.222 | 1.000 | .. | 0.231 |
| Tubular vs Papillary | 0.164 | 0.021-1.272 | 0.084 | 0.169 | 0.022-1.305 | 0.088 |
| Mucinous vs Papillary | 0.198 | 0.020-1.948 | 0.165 | 0.167 | 0.017-1.657 | 0.126 |
| Location |  |  |  |  |  |  |
| Sigmoid+Rectum | 1.000 | .. | 0.365 | 1.000 |  | 0.253 |
| Right hemicolon | 1.520 | 0.566-4.084 | 0.406 | 1.985 | 0.826-4.773 | 0.126 |
| Transverse colon | 0.000 | 0.000-.. | 0.982 | 0.000 | 0.000-.. | 0.982 |
| Left hemicolon | 2.906 | 0.893-9.449 | 0.076 | 2.911 | 0.908-9.333 | 0.072 |
| Dukes stage |  |  |  |  |  |  |
| Ⅳ | 1.000 | .. | **0.002** | 1.000 | .. | **0.001** |
| Ⅰ vs Ⅳ | 0.086 | 0.016-0.449 | **0.004** | 0.067 | 0.013-0.335 | **0.001** |
| Ⅱ vs Ⅳ | 0.194 | 0.064-0.593 | **0.004** | 0.161 | 0.056-0.460 | **0.001** |
| Ⅲ vs Ⅳ | 0.107 | 0.028-0.407 | **0.001** | 0.153 | 0.050-0.463 | **0.001** |
| Tumor size(＜5vs≥5,cm) | 0.537 | 0.219-1.314 | 0.173 | 0.550 | 0.244-1.240 | 0.149 |
| CEA(＜5vs≥5 ng/mL) | 1.289 | 0.527-3.154 | 0.578 | 1.042 | 0.467-2.328 | 0.919 |
| CXCL5(low vs high) | 0.229 | 0.067-0.783 | **0.019** | 0.165 | 0.049-0.556 | **0.004** |
| **Multivariate analysis** |  |  |  |  |  |  |
| Dukes stage |  |  |  |  |  |  |
| Ⅳ | 1.000 |  | **0.024** | 1.000 | .. | **0.021** |
| Ⅰ vs Ⅳ | 0.168 | 0.030-0.956 | **0.044** | 0.152 | 0.028-0.817 | **0.028** |
| Ⅱ vs Ⅳ | 0.277 | 0.089-0.863 | **0.027** | 0.248 | 0.085-0.720 | **0.010** |
| Ⅲ vs Ⅳ | 0.139 | 0.036-0.531 | **0.004** | 0.210 | 0.069-0.644 | **0.006** |
| CXCL5(low vs high) | 0.295 | 0.081-1.078 | 0.065 | 0.227 | 0.064-0.813 | **0.023** |

**Table S6** Pathological Stages of Patients in Figure 2A&B

| Case NO. | TNM | Dukes | Case NO. | TNM | Dukes | Case NO. | TNM | Dukes |
| --- | --- | --- | --- | --- | --- | --- | --- | --- |
| 1 | T4N1M0 | III | 11 | T4N0M0 | II | 21 | T2N1M0 | III |
| 2 | T4N0M0 | II | 12 | T4N0M0 | II | 22 | T3N0M0 | II |
| 3 | T4N2M0 | III | 13 | T4N2M0 | III | 23 | T3N1M0 | III |
| 4 | T4N1M0 | III | 14 | T4N2M0 | III | 24 | T3N0M0 | II |
| 5 | T2N0M0 | I | 15 | T4N1M0 | III | 25 | T4N0M0 | II |
| 6 | T3N2M0 | III | 16 | T3N0M0 | II | 26 | T4N2M0 | III |
| 7 | T2N0M0 | I | 17 | T3N0M0 | II | 27 | T2N0M0 | I |
| 8 | T4N0M0 | II | 18 | T4N1M0 | III | 28 | T3N1M0 | III |
| 9 | T3N0M0 | II | 19 | T2N0M0 | I | 29 | T2N0M0 | I |
| 10 | T3N0M0 | II | 20 | T3N0M0 | II | 30 |  |  |

**Supplementary Methods**

**Tissue microarray and immunohistochemical analysis**

Fresh specimens collected were immediately fixed by 4% formaldehyde after dissection and embedded with paraffin. Tissue microarray were manufactured by Shanghai Outdo Biotechnology Corporation. Immunohistochemical assay was performed as previously described(1). Details can also be acquired in Supplementary methods. Intensity of immunohistochemical staining of CXCL5 in tumour tissue was scored by two independent pathologists according to semi-quantitative immunoreactivity scoring (IRS) system(2). Intensity of immunostaining was scored as 0 (no immunostaining), 1 (weak immunostaining), 2 (moderate immunostaining) and 3 (strong immunostaining). The percentage of immunoreactive cells scoring was documented as 0 (none), 1 (<10%), 2 (10-50%), 3 (51-80%) and 4 (>80%). The intensity of immunostaining score and the percentage of immunoreactive cells score were multiplied to generate IRS ranging from 0 to 12 for each tumour. The optimum cut-off value 4.5 was calculated by using score X-tile software version 3.6.1 (Yale University School of Medicine, USA) based on the association with the patients’ overall survival. IRS more than or equal to 4.5 was regarded as high expression and less than 4.5 was regarded as low expression of CXCL5 (Supplementary Figure 2 ).

**Lentivirus vectors and shRNA transfection**

The lentiviral vectors LV5-EF1a-GFP/Puro-CXCL5, LV3-pGLV-h1-GFP/puro-shCXCL5, LV3-pGLV-h1-GFP/puro-shCXCR2, LV5-EF1a-GFP/Puro-NC and LV3-pGLV-h1-GFP/puro- NC were constructed by the Shanghai GenePharma Corporation (Shanghai, China). The sequences of shCXCL5 and shCXCR2 are shown in Supplementary Table 1. GFP-labeled short hairpin RNAs (shRNA) targeting Snail and β-catenin were purchased from the Shanghai GenePharma Corporation (Shanghai, China). The targeting site sequences are listed in Supplementary Table 1. The overexpression and interfering effects of these vectors/shRNAs were evaluated by immunoblot.

Lentivirus transfection was performed according to the manufacturer’s instruction. Briefly, 3×10^5^ cells were seeded into each well of six-well plate one day before transfection. When cells were in 70% confluence, change culture medium with fresh normal medium. For each well, 50 μL primary lentivirus solution was diluted by 400 μL normal medium and Polybrene was added at final concentration 5 μg/mL. Then the mixture was added into each well. After 24 hours, change the medium in each well. Forty-eight hours after transfection, antibiotics puromycin was used to screen stable cell clones. Protein levels of the target genes were assessed by immunoblot.

GFP-labeled short hairpin RNA(shRNA) targeting Snail and β-catenin were purchased from Shanghai GenePharma Corporation (Shanghai, China). As to transfection, 3×10^5^ cells were seeded into each well of six-well plate one day before transfection. When cells were in 70% confluence, LipofectamineTM 3000 (Invitrogen Corporation) was used to mediate shRNA against Snail or β-catenin into cells, shRNA targeting a non-homologous gene was performed as control. Cell culture medium was changed into FBS-free medium 6 hours before transfection and then 5 μg shRNA in 250 μL FBS-free medium and 10 μL Lipo3000 in 250 μL FBS-free medium were mixed uniformly before being added into each well. After 6 hours, 1 mL normal medium was added into each well. Twelve hours after transfection, medium was changed into fresh normal medium. Forty-eight hours after transfection, antibiotics puromycin(for lentivirus vector) or G418 (for plasmid vector) was used to screen stable cell clones. Protein levels of the target genes were assessed by immunoblot.

**Enzyme-Linked Immunosorbent Assay (ELISA)**

The level of CXCL5 in cell culture supernatants was detected using Human CXCL5/ENA-78 Quantikine ELISA Kit(R&D, USA). The experiment was performed according to the manufacturer’s instructions. In short, 200 µL of Assay Diluent was added to each well firstly and then 50 µL of standard, control, or sample was added to each well. The plate was covered with a plate sealer and incubated at room temperature for 2 hours. After being washed for 3 times, 200 µL of Conjugate was added to each well. Then incubate the plate at room temperature for 2 hours. After being washed for 3 times, 200 µL of Substrate Solution was added to each well. Then incubate the plate at room temperature for 30 minutes. And then add 50 µL of Stop Solution to each well. The absorbance was read at 450 nm within 30 minutes.

**RNA isolation and qRT-PCR**

Total RNA of frozen tissues was extracted using Trizol reagent(Invitrogen, CA) according to the manufacturer's instructions. Total RNA was reversed to cDNA using High Capacity cDNA Reverse Transcription Kit (ThermoFisher, USA) and qRT-PCR was performed using SYBR Green (ThermoFisher, USA) to detect the expression of CXCL5 mRNA in 29 pairs of tissue according to the manufacturer's instructions. GAPDH was used as control. The primers of CXCL5 and β-actin were purchased from Sangon Biotech, Shanghai and the sequences were listed as follows: CXCL5(5’-3’) Forward primer: GAGAGCTGCGTTGCGTTTGTTTAC; Reverse primer : CCGTTCTTCAGGGAGGCTACCA; β-actin(5’-3’) Forward primer : CGTGGGCCGCCCTAGG CACCA; Reverse primer : TTGGCTTAGGGTTCAGGGGGG. Relative mRNA levels were calculated and described as 2^-ΔCt^(ΔCt= Ct^CXCL5^- Ct^β-actin^) and 2^-ΔΔCt^(ΔΔCt=ΔCt^Tumor^-ΔCt^Normal^).

**Protein extraction and immunoblot analysis, cell invasion and migration assays**

Cells were collected at a 70-80% confluence and were lysed in RIPA (Solarbio, China) for 30 min on the ice. Then the total protein lysate was centrifuged for 20 min at 13,000 rpm. The supernatant was collected for immunoblot analysis. The concentration of total protein lysate was tested using BCA Protein Assay Kit (ThermoFisher, USA).

As to immunoblot analysis, equivalent amounts of protein were mixed with loading buffer (Bio-Rad, USA) and then added into each well of SDS-PAGE gel (10% or 12%). After electrophoresis, proteins were transferred from SDS-PAGE gel to PVDF membranes which were then blocked for 2 h in 5% nonfat dry milk or BSA in TBS. After blocking, the membranes were incubated with specific primary antibody at 4℃overnight. The primary antibodies and dilutions used in the immunoblots are listed in Supplementary Table 2. The membranes were then treated with HRP-conjugated secondary antibody(diluted at 1:5000) and the ECL chemiluminescence agent (Millipore, USA) was used to visualize the membrane. The image was captured by Tanon Chemiluminescence Imaging System (Tanon, China).

Cell invasion and migration assay were performed by using transwell chamber (8 mm, 24-well Format, Corning, USA) which was coated with or without diluted Matrigel (BD Biosciences). Assays were performed as following: 200 μL serum-free medium containing 3×10^5^ cells were placed in the upper chamber and 600 μL culture medium with 10% serum added into the lower chamber as attractant. The chamber was cultured in 37 °C , 5% CO_2_ incubator for 24 h. Cotton swab was used to remove redundant cells in the top chamber and then the chambers were fixed with methanol. Cells on the bottom of the chamber were stained with 1% crystal violet for 30 min. Cell number of 5 random fields were counted under 20×(objective lens) microscope and the results are described as means±SD.

**Immunofluorescence assays**

For the immunofluorescence assays, 40,000 cells were cultured in each well of EZ slides (Millipore, USA). After a 24-hour incubation, the cells in each well were fixed with 4% paraformaldehyde for 15 minutes. After rinsing with PBS, the cells were permeabilized with 0.1% Triton X-100 for 15 minutes at room temperature. The cells were then washed three times with PBS and blocked with 5% BSA for 1 hour at room temperature. The cells were incubated with the primary antibody at 4 ℃ overnight. After that, each well was washed 3 times with PBS and incubated with the iFluorTM 594 goat anti-mouse or rabbit antibody (AAT Bioquest, USA) for 2 hours at 37 ℃. After washing with PBS, diamidino phenylindole (DAPI, Santa Cruz, USA) was used to counterstain the nucleus. The results were visualized using a laser scanning confocal microscope (Zeiss, Germany). More information about the primary antibody is listed in Supplementary Table 3.

**References**

**1.** Zhao J, Li P, Feng H, Wang P, Zong Y, Ma J, et al. Cadherin-12 contributes to tumorigenicity in colorectal cancer by promoting migration, invasion, adhesion and angiogenesis. J Transl Med 2013; 11: 288-298.

**2.** Wilko Weichert, Annika Röske, Volker Gekeler, Thomas Beckers, Matthias P A Ebert, Matthias Pross, et al. Association of patterns of class I histone deacetylase expression with patient prognosis in gastric cancer: a retrospective analysis. Lancet Oncol 2008; 9:139-148.
